# Decision-Level Multi-Sensor Coordination for Robust Navigation and High-Precision Planar Positioning of Industrial Mobile Robots

**DOI:** 10.3390/s26175655

**Published:** 2026-09-05

**Authors:** Teng-Xiao Liu, Ming-Wei You, Zi-Yi Zhang, Yan Sun, Cheng-Yuan Liu, Kun Qian, Xue-Yu Lu

**Affiliations:** 1 School of Mechanical Engineering, Jiangnan University, Wuxi 214122, China; 1042230215@stu.jiangnan.edu.cn (T.-X.L.); 1042230201@stu.jiangnan.edu.cn (Z.-Y.Z.); 1044230523@stu.jiangnan.edu.cn (Y.S.); 1044220225@stu.jiangnan.edu.cn (C.-Y.L.); 2 School of Internet of Things Engineering, Jiangnan University, Wuxi 214122, China; 1036230116@stu.jiangnan.edu.cn; 3 School of Artificial Intelligence and Computer Science, Jiangnan University, Wuxi 214122, China

**Keywords:** edge computing, decision-level multi-sensor coordination, industrial mobile robot, robust navigation, planar positioning, visual servoing, YOLOv5s

## Abstract

High-precision manufacturing in unstructured factories imposes stringent requirements on real-time scene perception and end-effector positioning accuracy. Traditional single-sensor solutions suffer from perception blind spots in human-robot mixed environments with complex lighting, while chassis cumulative error often leads to rigid collisions during end-effector operations. To address this, this paper proposes and evaluates a decision-level multi-sensor coordination mechanism for robust navigation and high-precision planar positioning of industrial mobile robots. The mechanism assigns explicit sensor roles, distance-dependent trigger conditions, and deterministic safety priorities. At the navigation and obstacle avoidance level, a sequential decision policy is constructed: macroscopically, a lightweight You Only Look Once version 5 small (YOLOv5s) is utilized for the early detection of dynamic objects, providing bounding-box coordinates to trigger preemptive deceleration, while LiDAR independently provides geometric ranging for ROS local-costmap updating and detour replanning; microscopically, a low-level hardware interrupt strategy triggered by ultrasonic sensors is proposed to mitigate near-field blind spots and reduce communication latency. At the end-effector positioning level, under illumination conditions ranging from 200 to 1000 lux, an adaptive alignment algorithm combining hue-saturation-value color-space morphological processing and Kalman filtering is proposed to suppress measurement noise caused by illumination variations and mechanical vibrations. Experiments in the tested dynamic human-robot mixed scenarios showed no rigid collisions for the proposed system and an emergency response time of approximately 50 ms against sudden blind-spot intrusions. Simultaneously, the system achieves a 95% reliability rate in controlling the end-effector 2D planar positioning error (X-Y plane) within a ±2 mm tolerance under complex illumination interference. These results demonstrate improved navigation safety and planar-positioning reliability under the tested flexible-manufacturing conditions.

## 1. Introduction

Driven by the rapid evolution of intelligent manufacturing, intralogistics systems are transitioning from rigid automation to flexible intelligence [1,2]. As critical nodes in this ecosystem [3,4], industrial mobile robots (IMRs) are essential for connecting discrete production stages and optimizing operational efficiency. Although traditional automated guided vehicles operate effectively in structured settings [5,6,7], modern workshops typically exhibit highly dynamic and unstructured characteristics. Such environments are defined by mixed traffic, frequent human traversal, and variable lighting [8,9], posing severe challenges to the navigational safety and precise localization of IMRs.

The Robot Operating System (ROS) navigation stack has become the mainstream framework for mobile robot development [10,11]. However, traditional schemes relying solely on single-LiDAR (light detection and ranging) sensors face distinct adaptability limitations in complex industrial scenarios [12]. Recent studies have therefore emphasized multi-sensor mapping and intelligent path tracking algorithms for dynamic and unstructured environments. For instance, multimodal fusion architectures have been proposed to mitigate sensor degradation and improve terrain perception [13], while dynamic path planning and cooperative scheduling algorithms based on hybrid multi-objective optimization have reported improved performance in complex, multi-constrained terrains [14].

Despite these advances, perception methods that rely mainly on geometric features still suffer from semantic blind spots. Dynamic entities may be indiscriminately mapped into the *static_layer* under a static world assumption [15,16,17]. Furthermore, standard 2D LiDARs inherently fail to detect optically transparent materials, such as glass or acrylic panels, frequently found in modern factories due to specular reflection. Lacking semantic distinction between walking operators and fixed facilities, these systems frequently misinterpret dynamic scenes [18,19]. Such deficiencies may lead to invalid path planning or navigation deadlocks, where the robot becomes immobilized because no safe, collision-free path is perceived.

While multi-sensor fusion mitigates single-source limitations, it introduces a conflict between computational demands and real-time performance on resource-constrained edge devices [20,21,22,23]. Integrating visual sensors supplements semantic information, yet high-precision models like Transformers or You Only Look Once (YOLO) typically mandate high-performance GPUs [24]. On embedded industrial controllers, deploying such heavyweight architectures induces latency that compromises the determinism essential for industrial-grade control [25].

Visual perception is also constrained by illumination fluctuations typical of industrial sites [26,27]. Workshop environments frequently experience strong reflections and fluctuating illumination, with illuminance varying from 200 to 1000 lux. In advanced flexible manufacturing, mobile manipulators are increasingly deployed for complex assembly tasks. According to performance measurement guidelines for advanced mobile manipulators in assembly applications (e.g., NIST standards) [28], ensuring the success of high-precision material handling and non-destructive assembly requires the absolute positioning accuracy of the end-effector to be strictly controlled within ±2 mm. However, traditional single-sensor navigation schemes typically provide only centimeter-level accuracy. Under drastic illumination changes, traditional RGB-based visual servoing is highly susceptible to failure, causing severe perception drift and exceeding precision grasping tolerances [29,30,31]. Chassis-level centimeter errors can propagate along the kinematic chain to the end-effector, increasing the risk of rigid collisions or grasping failures. Therefore, reducing the accuracy gap between centimeter-level chassis navigation and millimeter-scale manipulator operation under complex disturbances remains a key bottleneck for IMR deployment in flexible manufacturing.

To address these challenges, this paper proposes and evaluates a decision-level multi-sensor coordination mechanism for robust navigation and high-precision planar positioning of industrial mobile robots. The ROS navigation stack provides the baseline path-planning and navigation functions, while the proposed coordination mechanism specifies how semantic recognition, geometric ranging, near-field protection, and visual servoing are activated and prioritized to compensate for the limitations of conventional navigation in dense human–robot traffic, transparent-obstacle avoidance, and centimeter-level chassis positioning during the final manipulation stage. The hardware–software arrangement provides a reproducible realization of the proposed policy, while the methodological contribution lies in the explicit mapping from sensing conditions to prioritized motion actions. Specifically, the mechanism coordinates semantic obstacle recognition, LiDAR-based geometric local replanning, ultrasonic near-field protection, and image-based visual servoing through defined trigger conditions, spatial operating intervals, and safety priorities, thereby reducing the accuracy gap between centimeter-level chassis mobility and millimeter-scale manipulator operation under the tested industrial conditions. The main contributions of this paper are summarized as follows:

- **A decision-level multi-sensor coordination mechanism is proposed and explicitly formulated.** Instead of concatenating raw measurements or performing Kalman-filter-based vision–LiDAR state estimation, the mechanism assigns complementary roles to independent semantic, geometric-ranging, and near-field-ranging outputs and maps them to motion decisions through deterministic trigger conditions and safety-priority rules. The method-level contribution is the explicit decision policy that determines which sensing output is used, when it is activated, and which action takes priority under different spatial and safety conditions. The policy is expressed through observable sensor states and deterministic rules, allowing the contribution to be examined through subsystem comparisons and ablation experiments rather than being attributed only to the physical integration of the robot platform. This formulation also enables the coordination strategy to be analyzed and reproduced independently of the specific hardware implementation while retaining compatibility with resource-constrained edge platforms.
- **A semantic-enhanced, multi-level sequential decision strategy is developed to improve obstacle avoidance under the tested dynamic and near-field blind-spot conditions.** Macroscopically, a lightweight vision-triggered preemptive deceleration mechanism is introduced to actively manage dynamic kinetic energy. Microscopically, rather than relying on standard software-level integration, an ultrasonic-triggered hardware interrupt strategy is employed. This design bypasses standard ROS communication latency and mitigates optical-sensor blind spots for transparent materials; in the acrylic-panel intrusion test, it achieved an average response time of 48.2 ±2.4 ms.
- **A deterministic visual-kinematic closed-loop algorithm resilient to illumination interference is designed.** To mitigate severe illumination variations and mechanical vibrations common in industrial sites, the system cascades adaptive morphological processing with Kalman filtering. Instead of open-loop alignment, this algorithm continuously feeds the smoothed coordinate deviation into the Mecanum inverse kinematics and uses three consecutive valid frames as the convergence criterion. In the tested placement trials, this closed-loop method kept 95% of planar positioning errors within a ±2 mm tolerance.

## 2. System Architecture

### 2.1. System Realization of the Decision-Level Multi-Sensor Coordination Mechanism

**Figure 1:**
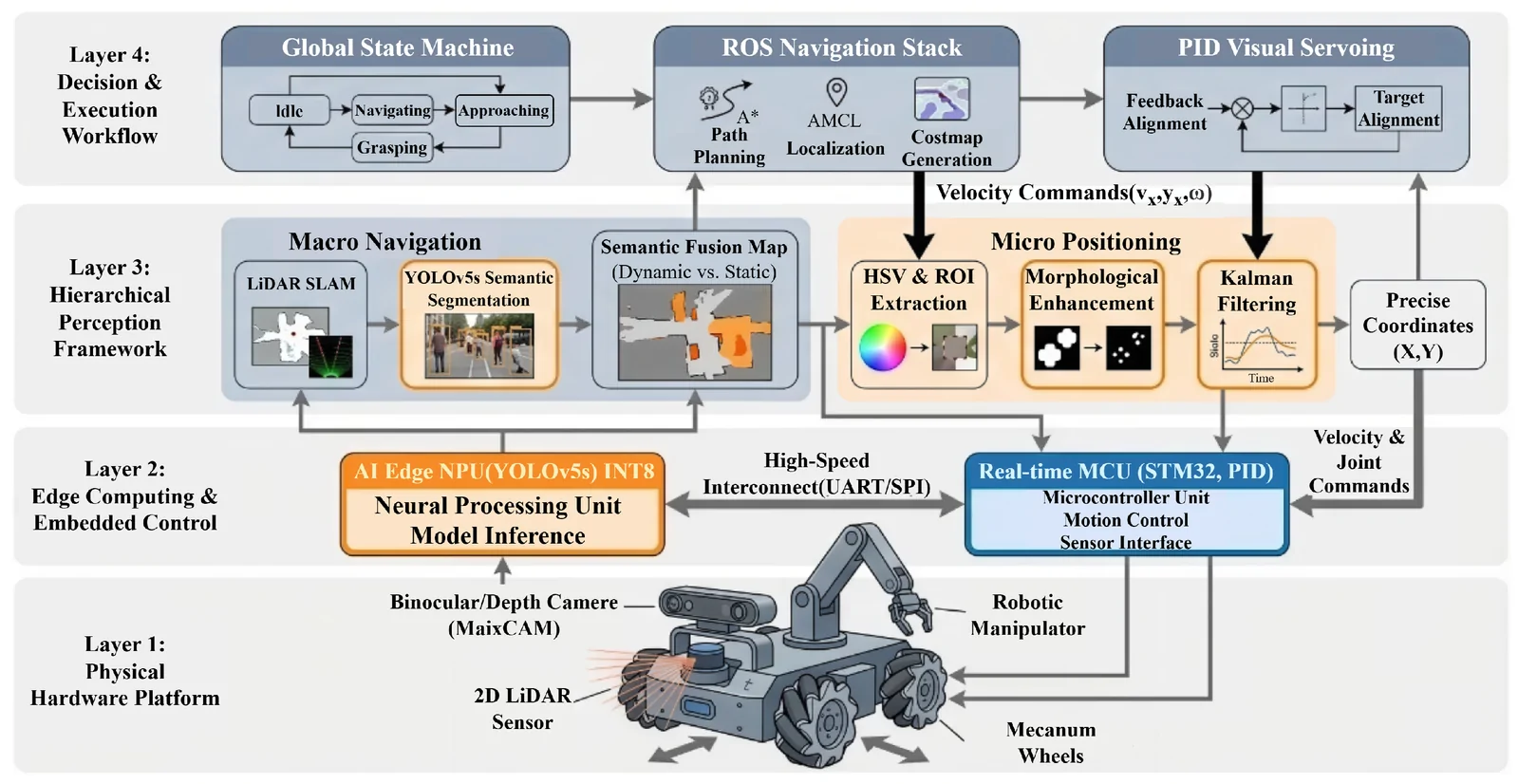
System realization of the decision-level multi-sensor coordination strategy for the industrial mobile robot. (A*: A-star algorithm; PID: proportional-integral-derivative; AMCL: adaptive Monte Carlo localization; ROI: region of interest; UART: universal asynchronous receiver-transmitter).

To address the perception and operational challenges in unstructured industrial environments, the proposed decision-level multi-sensor coordination mechanism is realized on the industrial mobile robot platform shown in Figure 1. The four logical layers organize the execution of the proposed decision policy from physical sensing to task-level action selection. The policy is defined by the sensor roles, spatial trigger conditions, and safety priorities described in Section 3.

The MaixCAM, based on the Sophgo CV181x processor, serves as the AI edge-computing node, while the low-level STM32 microcontroller unit (MCU) focuses on millisecond-level motion control and sensor data aggregation from the inertial measurement unit, ultrasonic sensor, and odometry. The MaixCAM and STM32 are decoupled through a high-speed universal asynchronous receiver-transmitter (UART) interface, allowing the visual perception and motion-control tasks to operate independently. Layers 3 and 4 realize the decision policy by assigning distinct roles to visual semantic recognition, 2D LiDAR geometric ranging, ultrasonic near-field sensing, and image-based visual servoing. The transition between macro navigation and micro manipulation is determined by explicit task states, spatial conditions, and safety priorities.

### 2.2. Physical Hardware Platform

**Figure 2:**
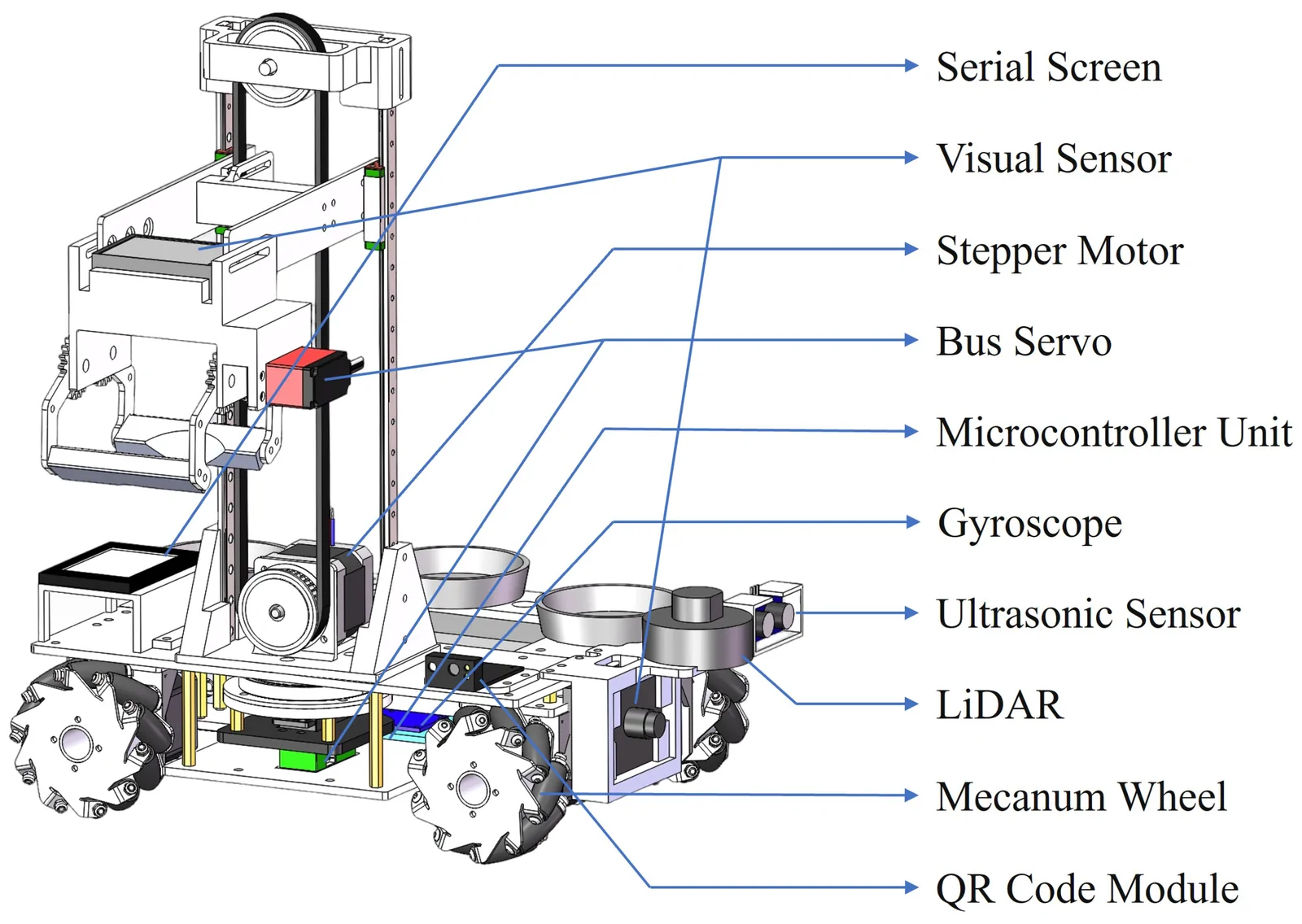
3D mechanical design of the IMR.

The physical structure of the proposed IMR is illustrated in Figure 2. The IMR is an in-house system jointly developed by the interdisciplinary research team, rather than a commercial product; its mechanical structure, embedded and electrical control hardware, visual-perception module, ROS-based navigation system, and software integration were developed in-house. The mobile base employs a four-wheel Mecanum layout. Compared to traditional differential drive systems, this configuration features omnidirectional mobility (x,y,θ), comprising translations along the two orthogonal planar axes *x* and *y* and yaw rotation θ, enabling zero-radius turning and lateral translation within narrow factory aisles. Kinematic resolution is based on a standard inverse kinematic model [32], which decomposes high-level velocity vectors into real-time angular velocity commands for the four motors.

Regarding the actuation mechanism, the system is equipped with a custom 3-Degrees-of-Freedom robotic manipulator. Unlike traditional lead screw or hydraulic structures, this manipulator utilizes a synchronous belt transmission system. This design reduces the inertia of the end-effector and is intended to reduce the influence of mechanical backlash during high-load vertical lifting. Its positioning performance is evaluated through the physical X–Y placement measurements reported in Section 4.6.

### 2.3. Network Communication and Remote Control Framework

**Figure 3:**
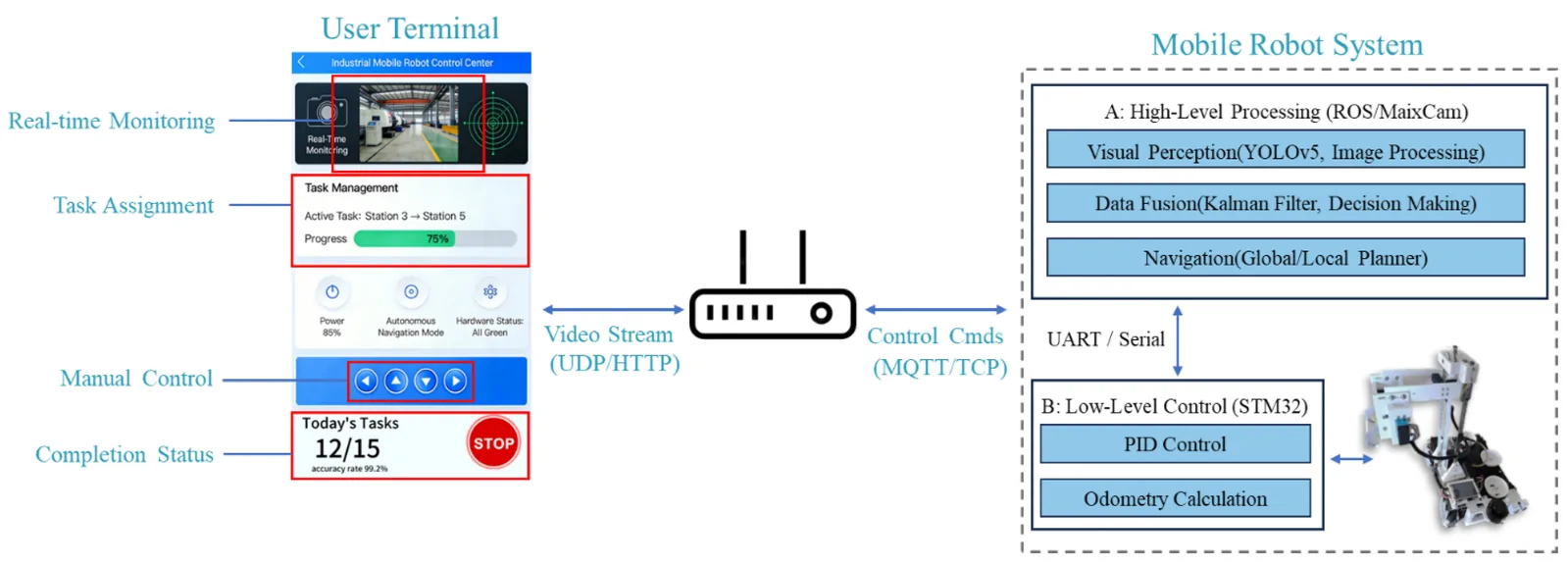
Network communication architecture and remote control framework. (UDP: user datagram protocol; HTTP: hypertext transfer protocol; MQTT: message queuing telemetry transport; TCP: transmission control protocol).

To ensure the efficiency and safety of human-robot interaction, the system establishes a dual-channel communication framework based on industrial Internet of Things standards, as shown in Figure 3. The onboard MaixCAM and STM32 controllers communicate through a UART serial interface, while Wi-Fi connectivity supports remote task interaction and status monitoring.

The system abandons single-protocol transmission in favor of a strategic traffic splitting mechanism:

1. **Command Stream:** Utilizing the message queuing telemetry transport protocol to transmit task scheduling, status telemetry, and motion commands. Its lightweight publish/subscribe mechanism ensures high reliability of control commands even in weak network environments.
2. **Video Stream:** Utilizing the user datagram protocol and hypertext transfer protocol to transmit real-time monitoring footage captured by the MaixCAM, prioritizing low latency and high throughput for the video feed.

Through this framework, the user terminal achieves remote task assignment and real-time status monitoring. Simultaneously, the onboard ROS navigation stack retains local autonomous obstacle avoidance authority, ensuring intrinsic safety during network fluctuations or disconnections.

## 3. Decision-Level Multi-Sensor Coordination Strategy

The proposed decision-level multi-sensor coordination mechanism is specified as an ordered safety policy with explicit sensing roles, spatial trigger conditions, and action priorities. In this study, coordination does not mean raw-data concatenation or Kalman-filter-based vision–LiDAR state-estimation fusion. Instead, the vision, LiDAR, and ultrasonic modules generate independent semantic, geometric-ranging, and near-field-ranging outputs, respectively; their complementary functions are coordinated through deterministic decision rules that select motion commands according to the active sensing condition and safety priority. In the navigation-safety chain, vision provides an image-plane semantic trigger for early deceleration, LiDAR provides the geometric ranging input used for local costmap updating and detour replanning, and ultrasonic sensing provides an overriding near-field emergency-stop signal. The Kalman filter described in Section 3.3 is outside this navigation-fusion chain: it smooths HSV/morphology-derived image-plane marker coordinates during local visual–kinematic alignment and does not receive or fuse LiDAR measurements.

**Figure 4:**
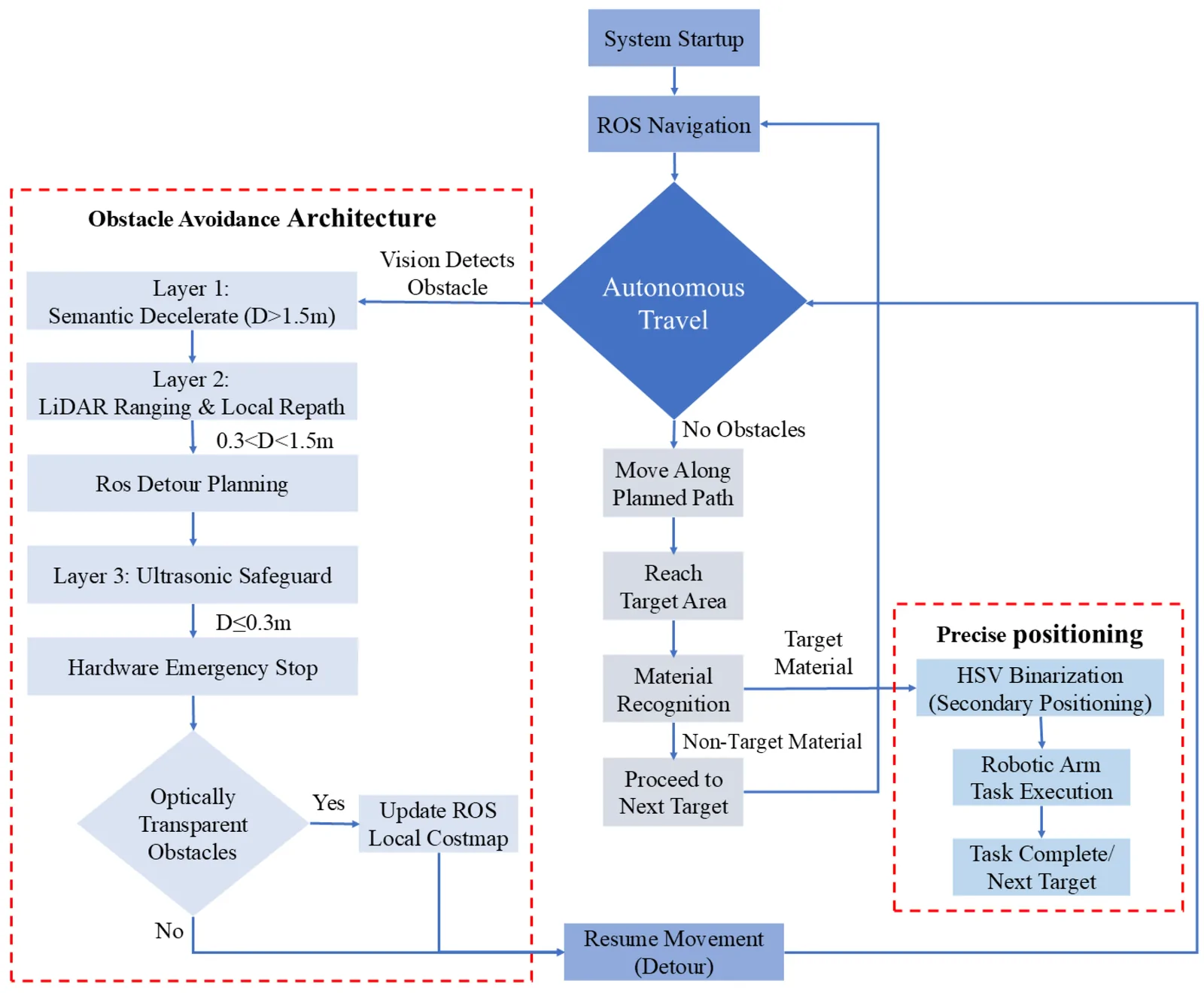
Schematic diagram of the decision-level multi-sensor coordination policy within the hierarchical safety sequence for progressive obstacle avoidance.

Figure 4 defines the proposed decision-level multi-sensor coordination policy within the hierarchical safety sequence for progressive obstacle avoidance. Each sensing modality is first processed by its own perception module; fusion occurs at the decision level, where deterministic rules select a safety action from the independently generated semantic, geometric, and near-field-ranging outputs. Thus, the model neither concatenates raw vision and LiDAR data nor uses a Kalman filter for vision–LiDAR state estimation. The onboard YOLOv5s module operates under a dual-task paradigm: it performs macro-level dynamic-obstacle detection during navigation and four-class workpiece recognition during precision manipulation.

For navigation safety, the deterministic decision rules shown in Figure 4 are defined as follows:

- **Vision-triggered preemptive deceleration layer (early-warning regime):** YOLOv5s provides semantic obstacle information and functions as a two-dimensional image-plane trigger; it does not estimate obstacle depth. When the center of a detected dynamic-obstacle bounding box enters the predefined image-plane proximity zone, the STM32 MCU issues a preemptive deceleration command to reduce chassis kinetic energy before a close-range encounter. Here, D>1.5 m is the LiDAR-measured distance condition, whereas the bounding-box center is used only to trigger the two-dimensional visual early warning; no visual depth estimation is involved.
- **LiDAR-driven geometric replanning layer (0.3 m <D≤1.5 m):** LiDAR continuously supplies geometric ranging information to the ROS navigation stack. When an obstacle enters the mid-range threshold, the system updates the local costmap and replans a detour path. Therefore, LiDAR contributes to geometric navigation and local path replanning; it is not used merely to trigger stopping.
- **Ultrasonic hardware-interrupt layer (D≤0.3 m):** The ultrasonic array provides a near-field safeguard against transparent or low-profile obstacles that may remain difficult for optical sensors. If the absolute safety threshold is breached, a highest-priority hardware emergency-stop command overrides all motion commands and bypasses the standard ROS communication pipeline.

The three layers form a parallel-sensing policy with state-dependent decision priorities: vision supplies semantic early warning, LiDAR supplies the geometric basis for local replanning, and ultrasonic sensing provides the final safety override. All three sensing modalities operate continuously; the distance intervals define the conditions under which the output of each modality has decision priority for its corresponding safety action. The novelty of this policy lies in explicitly mapping the complementary observability of the three modalities to differentiated control functions, rather than treating them as alternative sensors for the same control task. The Kalman filter used for HSV-based local visual alignment is not part of this decision policy.

The two distance thresholds were defined as action-priority boundaries and were subsequently examined through the one-factor sensitivity evaluation reported in Section 4.5.2. The 0.3 m ultrasonic threshold was selected as a conservative near-field emergency boundary. In the controlled acrylic-panel intrusion test at an initial chassis velocity of 0.5 m/s, which exceeded the normal maximum operational velocity of 0.4 m/s, the measured mean response time was 48.2±2.4 ms and the mean physical braking distance was 1.42±0.08 cm. The selected threshold therefore retained a substantial near-field margin relative to the measured stopping requirement. The 1.5 m upper decision boundary was selected to initiate vision-triggered preemptive deceleration sufficiently early for obstacle verification and, when required, ultrasonic point injection, local-costmap updating, and local replanning before the IMR reaches the close-range emergency region. Here, $D_{\mathrm{vis}}$ denotes the upper decision boundary associated with vision-triggered deceleration; it is not a monocular depth estimate, because the physical distance *D* is continuously supplied by the ranging sensors.

Within this hierarchical framework, the perception mechanism fundamentally decouples semantic recognition from geometric ranging to ensure real-time performance on edge devices. By avoiding computationally expensive monocular depth estimation, which inherently suffers from scale ambiguity, the vision module acts strictly as a 2D spatial trigger. The 2D LiDAR measures geometric ranges only within its fixed horizontal scanning plane at the mounting height, whereas the forward-facing camera provides appearance-based observation over a vertical field of view. Therefore, a structure that is insufficiently intercepted by the LiDAR scanning plane, such as a hollow steel frame with cross-members located above or below the scanning height, may still generate a semantic early-warning cue in the visual stream. Conversely, because this visual cue does not provide reliable metric depth, LiDAR remains responsible for range-based local-costmap updating and detour replanning. Concurrently, the absolute physical distance *D* is continuously and independently quantified by the parallel LiDAR and ultrasonic sensors. Thus, the condition D>1.5 m designates the macroscopic spatial interval where this vision-based early warning initiates, managing dynamic hazards without relying on visual depth measurements.

To prevent a navigation deadlock following an emergency stop, the MCU subsequently uploads the ultrasonic ranging data to update the ROS local costmap, artificially marking the transparent obstacle. This enables ROS to generate an effective detour path and safely resume movement.

This flowchart illustrates the complete decision-making process of the IMR, encompassing navigation, obstacle avoidance, workpiece recognition, and precision operation. It highlights the collaborative mechanism between multi-sensors (vision, LiDAR, ultrasonic) and algorithms such as ROS navigation, YOLOv5s recognition, and hue-saturation-value (HSV) binarization.

### 3.1. Visual Obstacle Avoidance

The experiment used a front-facing MaixCAM camera mounted on the IMR to acquire obstacle images in an indoor industrial experimental environment. A total of 1237 images were collected and divided into 954 training images, 140 validation images, and 143 test images. To represent variations relevant to practical obstacle-avoidance tasks, the dataset included changes in obstacle position, number, and partial occlusion, including single-obstacle, multiple-obstacle, and partially overlapping configurations. Images were collected under normal indoor illumination, local shadow conditions, and backgrounds with different colors and textures, including workbench surfaces and surrounding equipment areas. All images were manually annotated using rectangular bounding boxes. The annotations were subsequently reviewed for category consistency, bounding-box completeness, and bounding-box location accuracy; samples with ambiguous boundaries, severe occlusion, or uncertain category assignments were re-annotated or excluded before finalizing the dataset. YOLOv5s was trained for single-class obstacle detection using an input resolution of 448×448.

**Figure 5:**
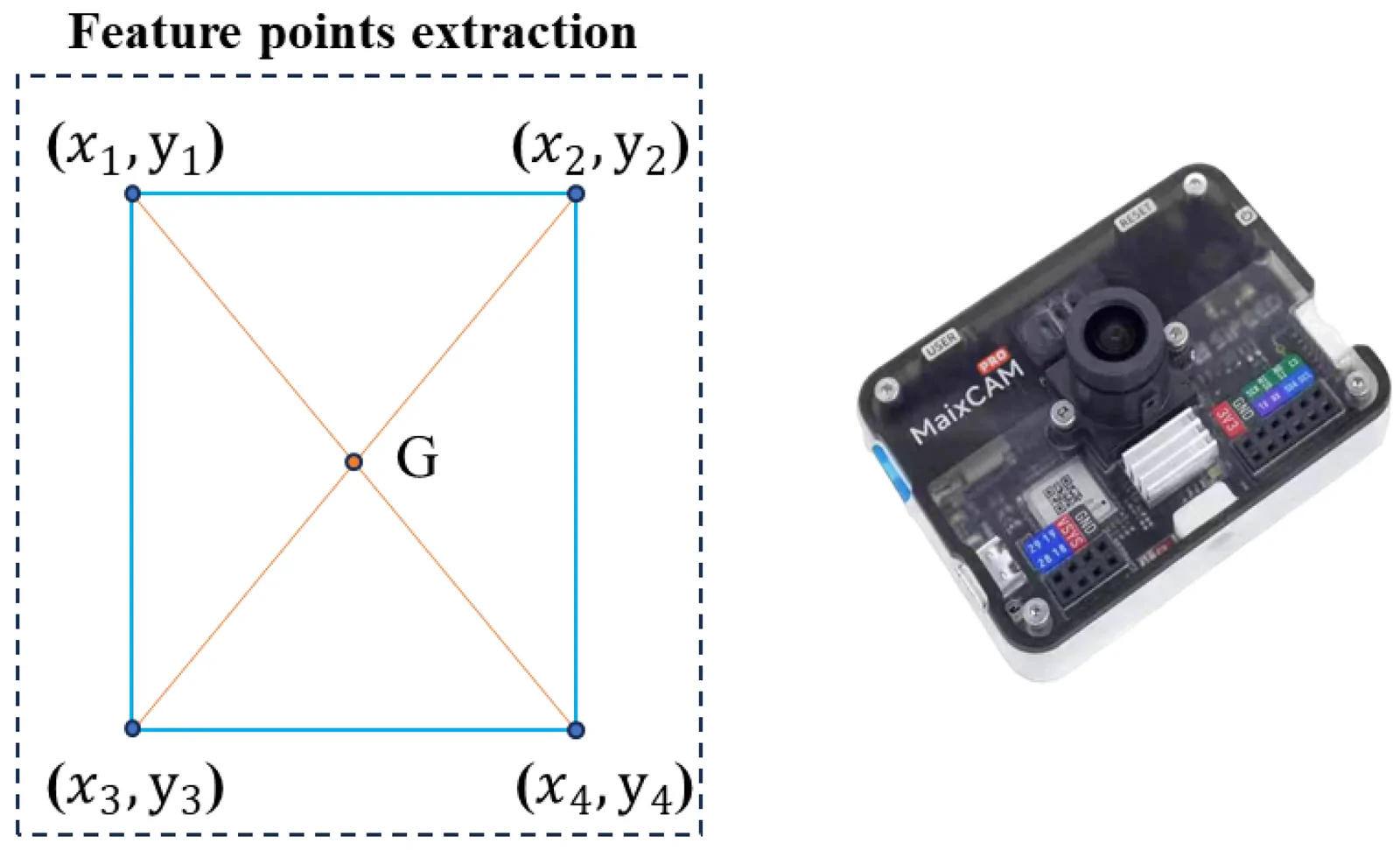
Obstacle bounding box localization.

As shown in Figure 5, when the front-facing camera of the IMR detects an obstacle, the real-time data is promptly fed back to the MCU. Through the ESP32 WiFi auxiliary chip, the images and recognition data are uploaded to the cloud platform. This allows users to view the specific operating status of the machine on the WeChat mobile interface in a timely manner and monitor the situation in front of the vehicle via real-time image transmission technology.

Simultaneously, the visual module establishes a two-dimensional planar coordinate system with the image center as the origin [33]. It acquires the coordinates of the four corner points of the obstacle bounding box, denoted as $\left(x_{1},y_{1}\right)$, $\left(x_{2},y_{2}\right)$, $\left(x_{3},y_{3}\right)$, and $\left(x_{4},y_{4}\right)$. The center point *G* of the bounding box is then located, and its coordinates are recorded and designated as the obstacle center coordinates.

### 3.2. Edge-Adapted YOLOv5s Detector for MaixCAM Deployment

To satisfy the real-time constraints of navigation safety and manufacturing operations, this study employs an edge-adapted lightweight YOLOv5s detector for semantic obstacle detection and workpiece recognition. The deployment adaptation targets the computational and runtime constraints of the MaixCAM platform and is evaluated in terms of recognition performance, inference speed, and runtime memory consumption. The obstacle-detection and workpiece-recognition tasks use the same lightweight network architecture, but are trained and evaluated using task-specific datasets and class configurations. This subsection describes the network architecture, model compression, and hardware-specific deployment adaptation.

#### 3.2.1. Network Architecture and MaixCAM Deployment Adaptation

**Figure 6:**
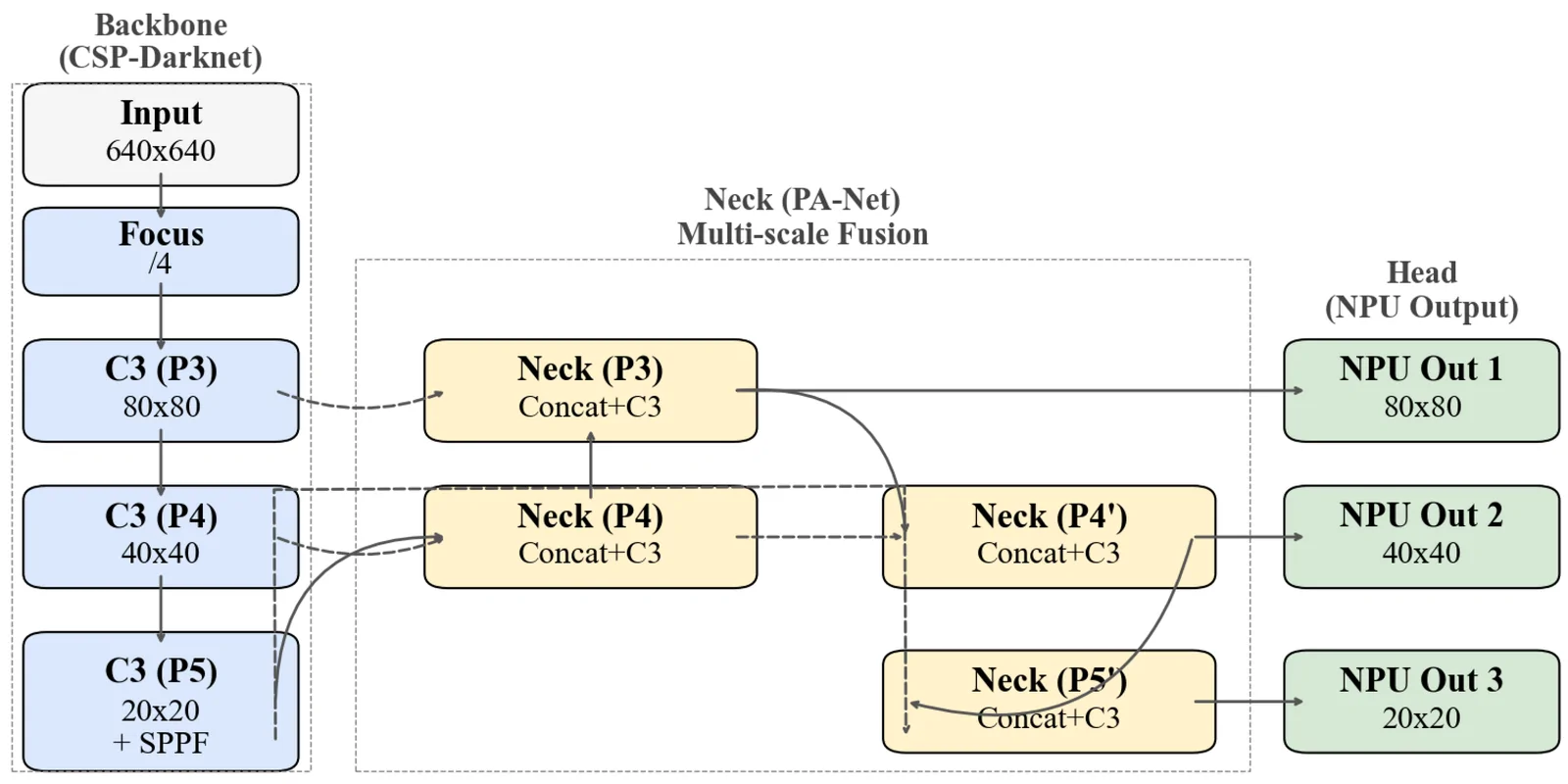
Architecture of the edge-adapted YOLOv5s detector deployed on MaixCAM.

As shown in Figure 6, the deployed YOLOv5s detector comprises a CSP-Darknet backbone, a PANet neck, and an INT8-compatible detection head. During feature extraction, the backbone generates a three-level feature pyramid, denoted as P3, P4, and P5, while the SPPF module enlarges the receptive field. The neck combines the feature pyramid network (FPN) and the path aggregation network (PAN) to propagate semantic information from deeper layers and reinforce localization information through bottom-up aggregation, thereby supporting multi-scale object detection.

The model was adapted to the NPU-supported inference pipeline of the Sophgo CV181x processor and converted through the platform toolchain for deployment on MaixCAM. In the deployed pipeline, supported convolution and tensor operations are executed by the on-chip NPU, whereas anchor decoding and non-maximum suppression (NMS) are performed on the CPU. Figure 6, illustrates the shared network architecture, while the task-specific detection categories are defined by the corresponding training annotations and deployment configuration.

#### 3.2.2. Edge-Deployment Performance Evaluation

**Figure 7:**
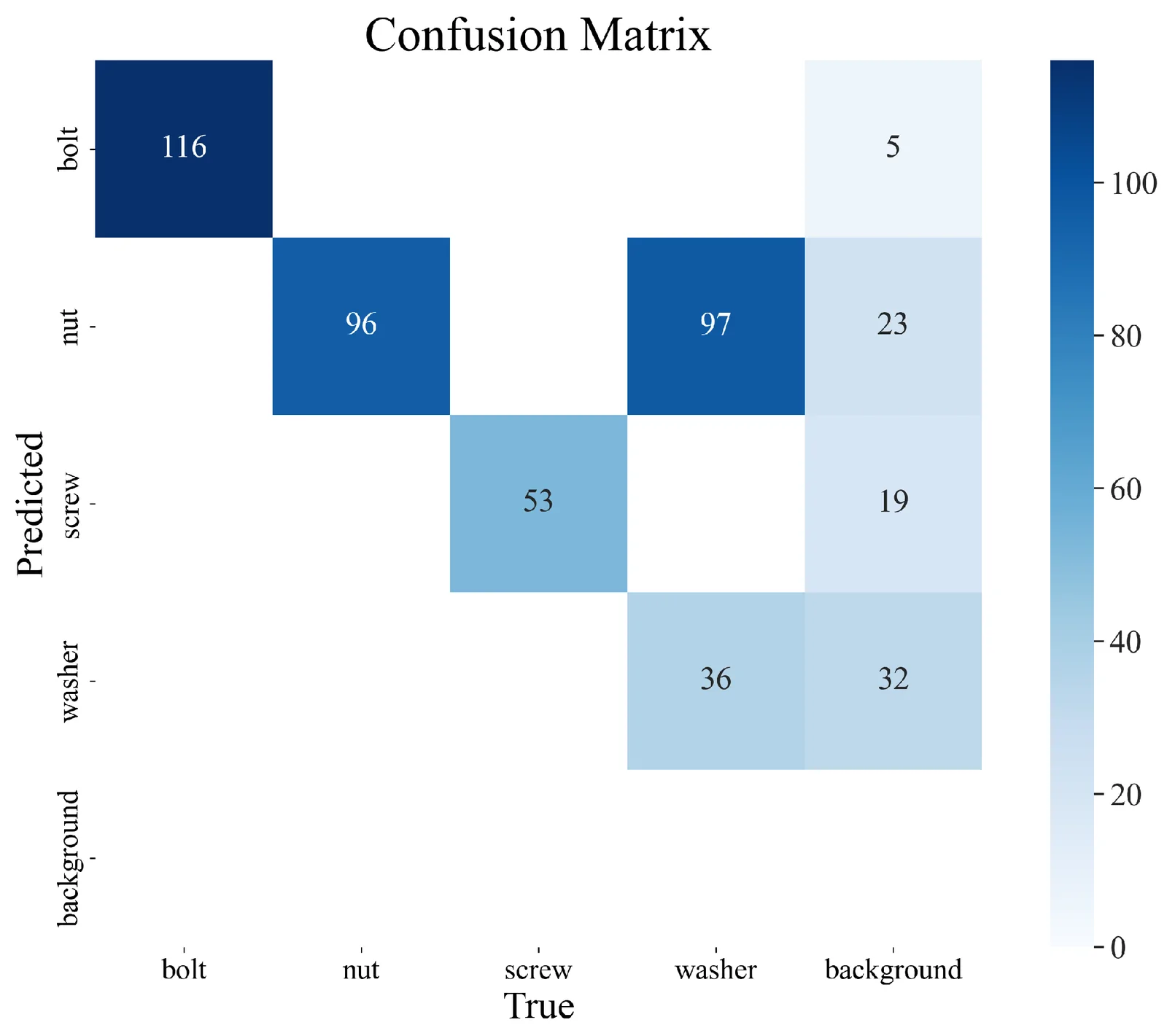
Raw confusion matrix of the INT8-deployed model for four-workpiece detection on the MaixCAM.

**Figure 8:**
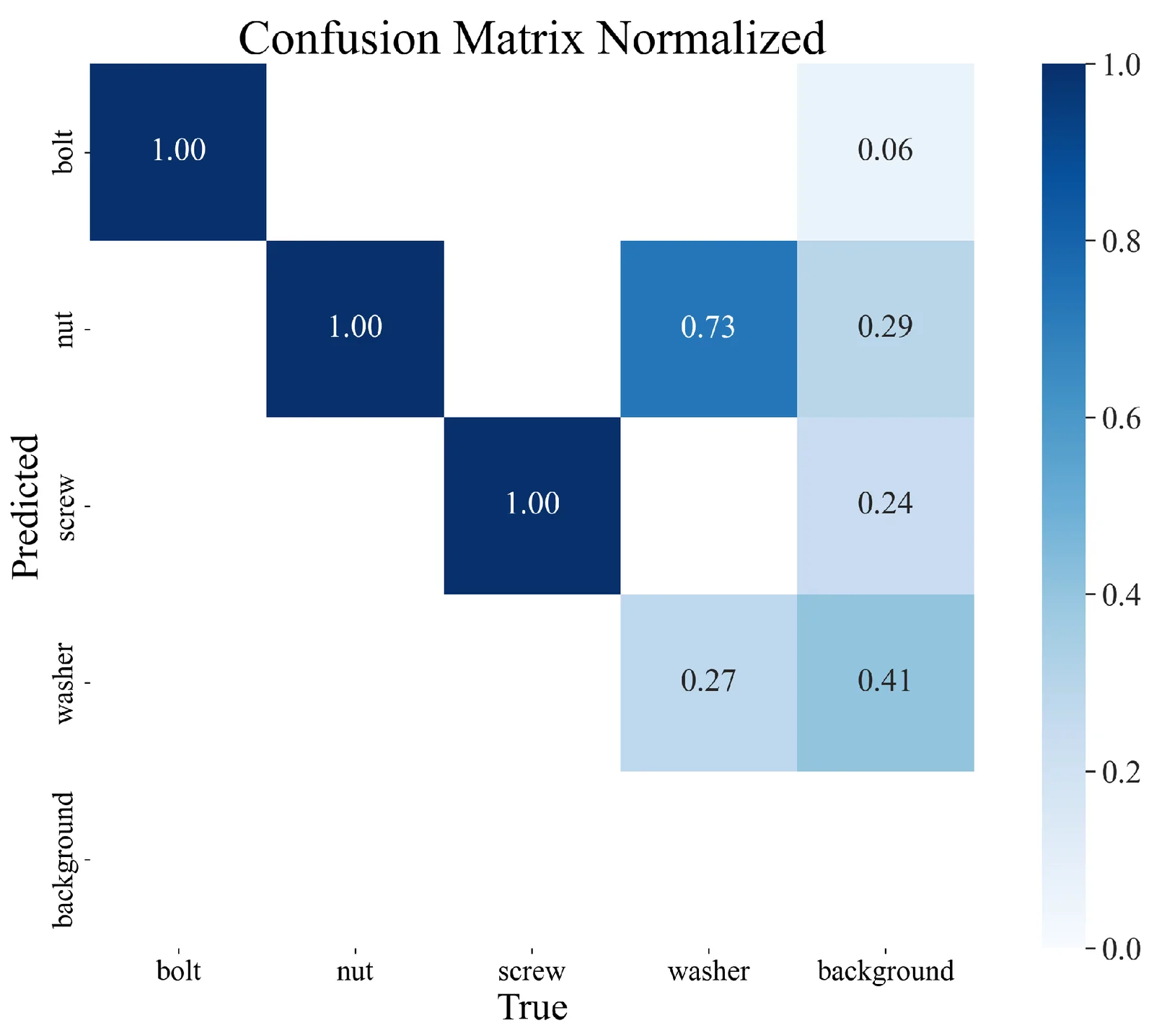
Normalized confusion matrix of the INT8-deployed model for four-workpiece detection on the MaixCAM.

To evaluate the actual edge-deployment performance for the four workpiece categories, raw and normalized confusion matrices were generated using the pruned and INT8-quantized model deployed on the MaixCAM, as shown in Figure 7 and Figure 8. In both matrices, columns represent ground-truth labels and rows represent predicted labels. The raw confusion matrix reports the detection counts, whereas the normalized matrix presents the prediction distribution for each ground-truth category. The four workpiece categories are bolt, nut, screw, and washer. The diagonal entries show that the bolt, nut, and screw instances were primarily assigned to their corresponding categories, with 116, 96, and 53 correctly predicted instances, respectively.

In contrast, the washer category presents a more challenging case: 36 washer instances were correctly predicted as washers, whereas 97 washer instances were predicted as nuts. This result indicates a class-dependent error pattern, with the distinction between nut and washer requiring further improvement under the present dataset configuration. The background row denotes ground-truth workpieces that were not matched by a prediction and therefore represents missed detections, while the background column denotes predicted boxes that were not matched to a ground-truth workpiece and therefore represents unmatched predictions. The background column contains 5, 23, 19, and 32 unmatched predictions assigned to bolt, nut, screw, and washer, respectively, providing a separate indication of the false-positive distribution among the predicted categories. Accordingly, the confusion matrices confirm effective recognition of several workpiece categories while identifying nut–washer confusion as the principal error mode to be addressed in subsequent optimization.

**Figure 9:**
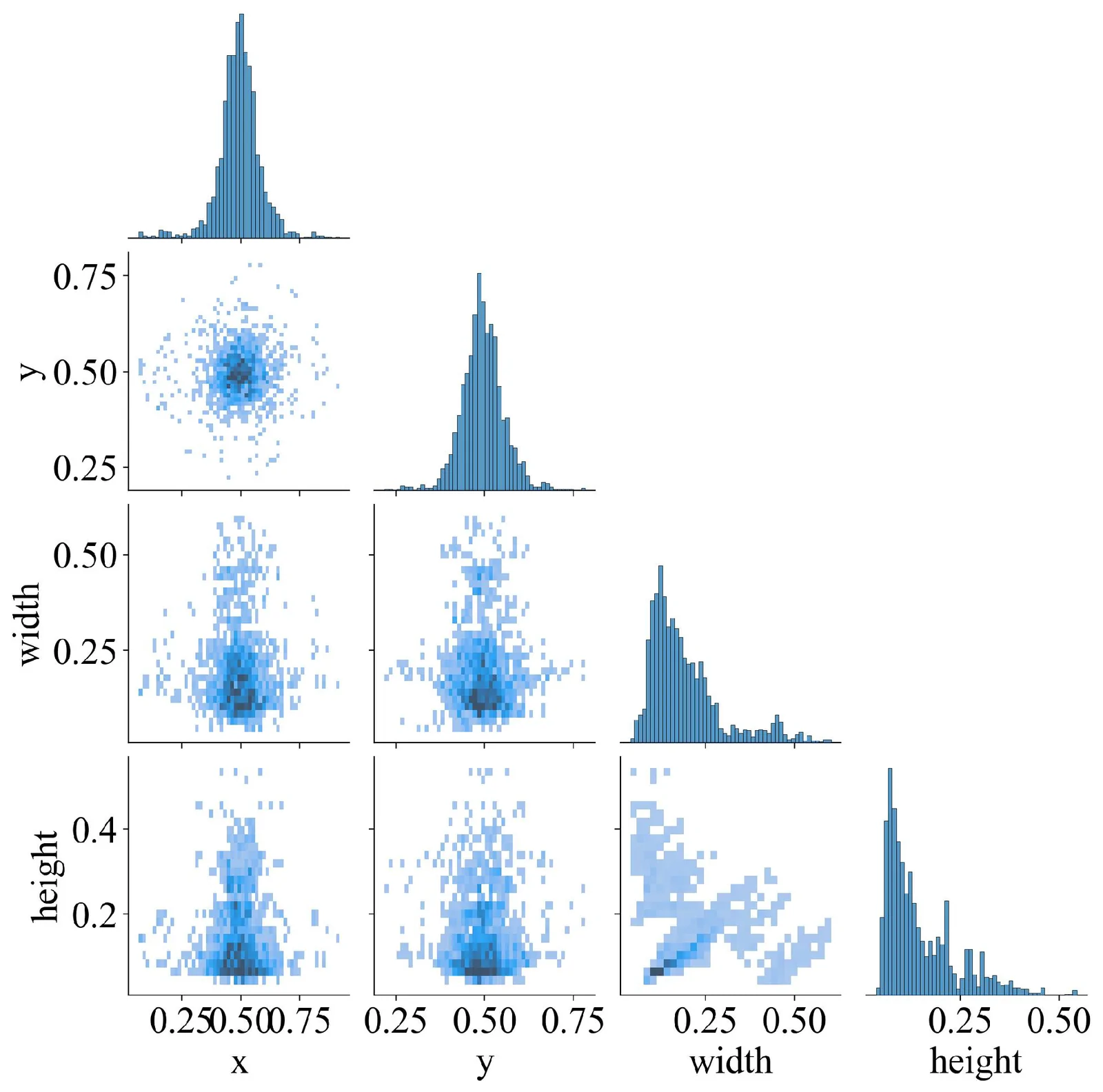
Labels correlogram of training annotations for four workpiece categories.

As shown in Figure 9, the spatial distribution and scale characteristics of the training annotations were analyzed using a labels correlogram. The figure displays the marginal distributions and pairwise relationships of the normalized bounding-box center coordinates (x,y) and dimensions (width,height). The histograms and heatmaps for *x* and *y* indicate the positional distribution of annotated workpieces in the image plane. Most targets are concentrated around the central region, while some occur at off-center positions. This observation serves as a diagnostic of the data-acquisition and annotation distribution and indicates a possible central concentration of the dataset; it does not by itself establish improved robustness to background interference.

The width and height distributions describe the range of target scales and aspect ratios represented in the training annotations. Together with the width–height scatter plot, these statistics are used to identify potential imbalances in target position, scale, or shape. YOLO training uses axis-aligned rectangular bounding boxes that enclose the visible extent of each workpiece. Therefore, irregular external appearances or contours of bolts, nuts, screws, and washers do not conflict with the rectangular annotation format used for detection training.

Figure 9 and the associated histograms and heatmaps are used solely to diagnose the annotation distribution of the four-class workpiece-recognition dataset. They are not inputs to the HSV-based local visual-alignment pipeline and do not provide evidence for the final X–Y planar positioning accuracy. The latter is evaluated independently in Section 4.6 through end-to-end physical placement measurements and module-ablation experiments.

### 3.3. Robust ROI Extraction and Dynamic Tracking

#### 3.3.1. Feature Restoration Based on HSV and Morphological Closing

To address RGB segmentation failures caused by illumination fluctuations under the tested 200–1000 lux conditions, HSV thresholding was adopted because it separates hue from saturation and brightness. Normalized RGB reduces intensity dependence but remains sensitive to localized specular reflections, whereas CIELAB requires computationally intensive non-linear color transformations on the embedded platform. YCbCr separates luminance from chrominance but retains coupling between color purity and tint that complicates threshold selection. In contrast, HSV enables hue-based thresholding of the target color under shadows and illumination variation while maintaining low conversion complexity. Under the tested illumination conditions, this representation was used to reduce the influence of specular reflections during marker segmentation.

**Figure 10:**
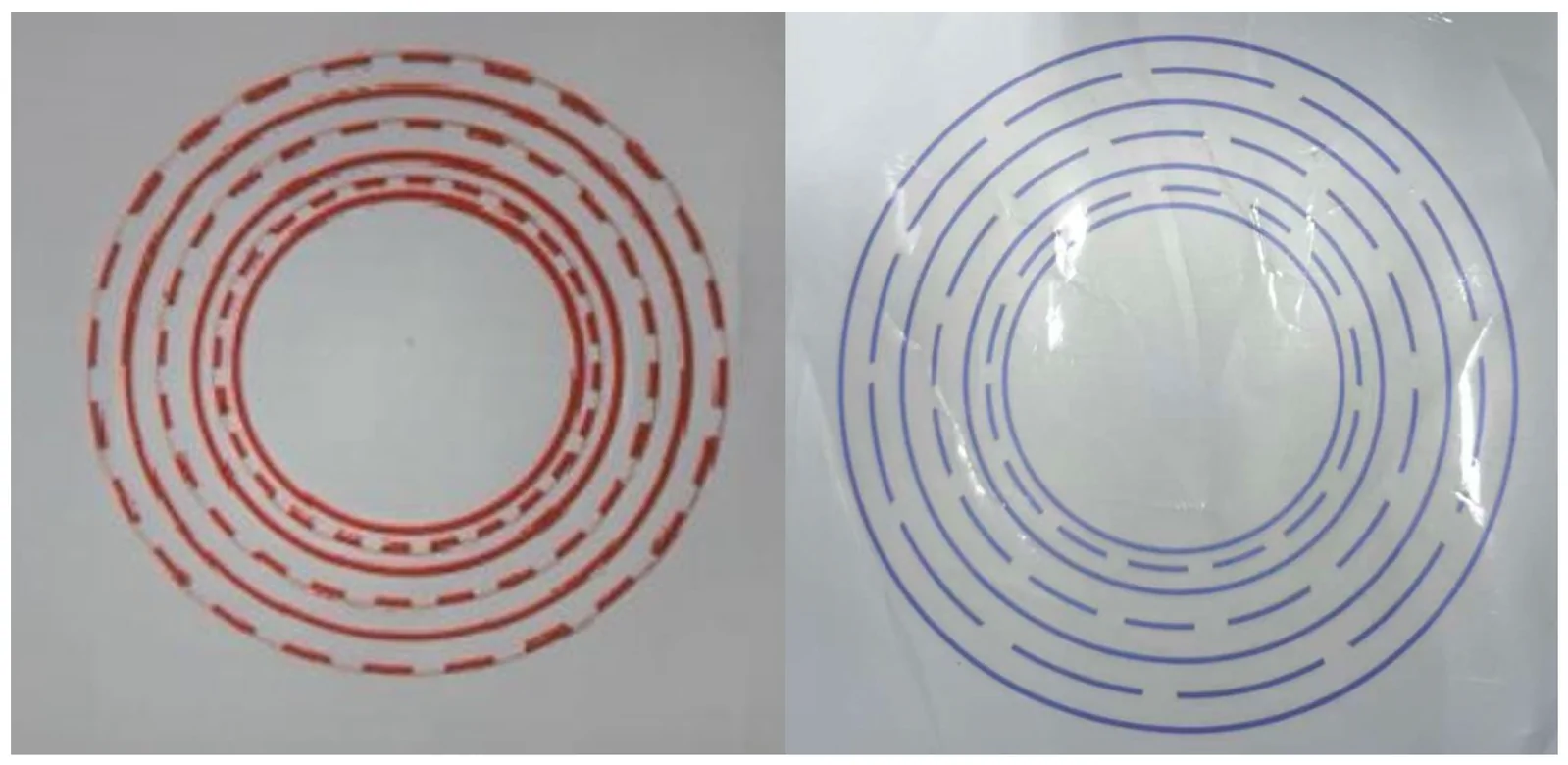
Unprocessed target rings under varying brightness and color conditions.

As shown in Figure 10, the discontinuous structure of the positioning markers produces disconnected arc segments in the initial binary image, which prevents the conventional Hough Circle Transform from extracting stable center coordinates. Therefore, a morphological closing operation, consisting of dilation followed by erosion, was applied to connect discrete feature points and restore a connected marker boundary before center extraction.

**Figure 11:**
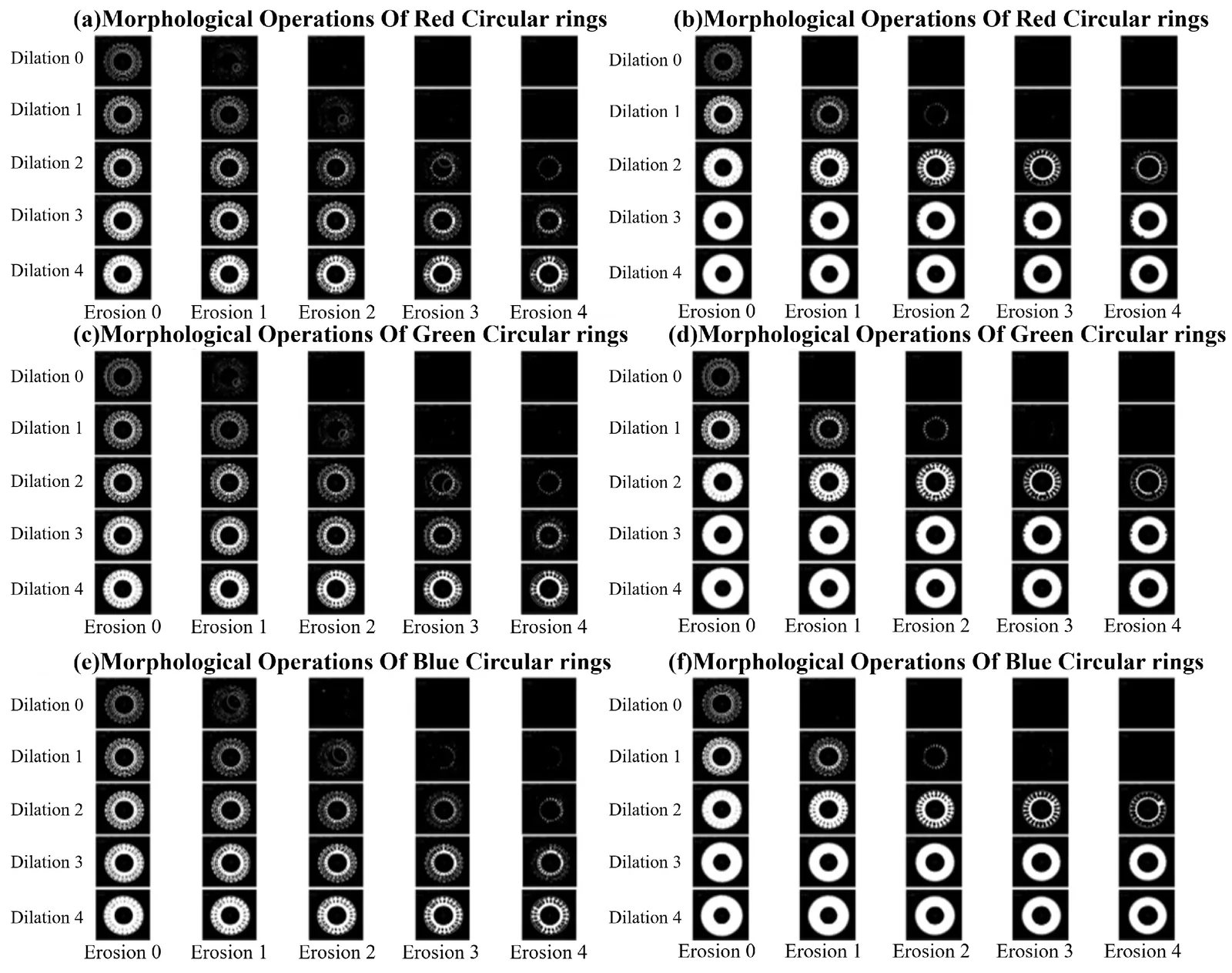
Parameter ablation matrix of morphological operations for RGB target rings under varying dilation and erosion iterations: (**a**,**b**) red circular rings; (**c**,**d**) green circular rings; (**e**,**f**) blue circular rings.

**Figure 12:**
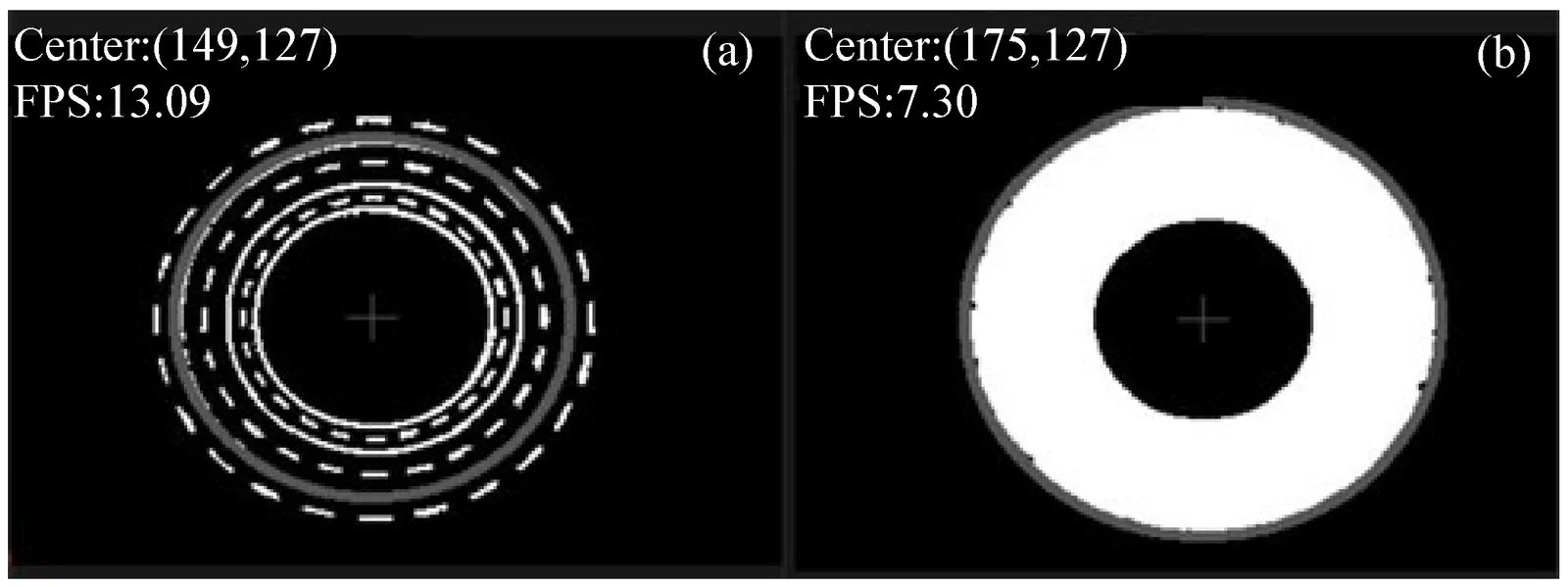
Comparison of target rings before and after processing: (**a**) image without closing operation; (**b**) image processed by the closing operation with appropriate parameters.

To select the morphological configuration, 3×3 and 5×5 structuring elements with dilation and erosion iterations ranging from 0 to 4 were evaluated, yielding 150 parameter combinations. Figure 11 shows that larger kernels or additional iterations improve contour continuity but increase the computational burden. A 5×5 rectangular structuring element with three dilation iterations and one erosion iteration was therefore selected as a balance between contour continuity and real-time operation. As illustrated in Figure 12, this configuration reconnects fragmented ring regions and suppresses isolated pixel artifacts, providing stable geometric input for center extraction.

#### 3.3.2. Visual Servoing Noise Suppression via Kalman Filtering

The six-state constant-acceleration Kalman filter is used only for local visual–kinematic X–Y alignment and is not part of the navigation coordination policy. It estimates the image-plane state ${\left[x,y,v_{x},v_{y},a_{x},a_{y}\right]}^{T}$ from HSV/morphology-derived marker coordinates. Before each local visual-alignment trial, yaw is corrected using the HWT101 IMU and a yaw PID loop until the absolute yaw error relative to the task-specific target angle is no greater than 0.2°; the subsequent visual controller performs only X–Y translational micro-adjustment.

**Figure 13:**
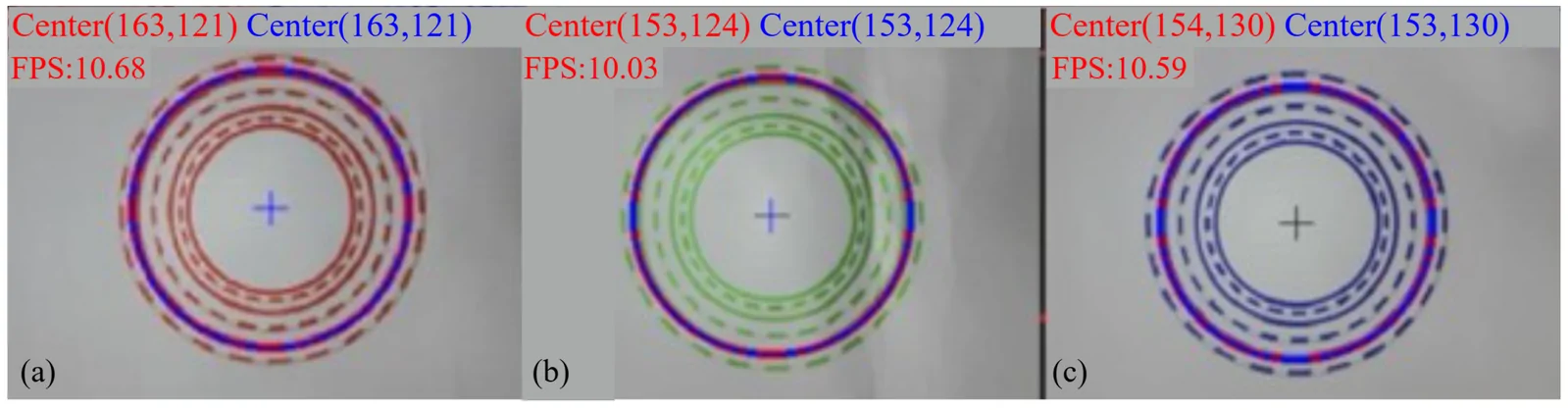
Comparison of contour extraction results before and after Kalman filtering, showing stabilized ring-center estimation: (**a**) red ring; (**b**) green ring; (**c**) blue ring.

During dynamic grasping by the robotic arm, chassis motion and mechanical vibrations introduce Gaussian white noise into the visual feedback, causing severe jitter in the calculated center coordinates (as shown by the red trajectory in Figure 13). To achieve smooth visual servoing control, the system introduces a Kalman Filter to construct a dynamic prediction model.

To suppress the measurement noise caused by mechanical vibrations and chassis jitter, a six-dimensional discrete Kalman filter based on a Constant Acceleration (CA) model is implemented. The state vector at time step *k* is defined as $x_{k}={\left[x_{k},y_{k},v_{x,k},v_{y,k},a_{x,k},a_{y,k}\right]}^{T}$, which denotes the position, velocity, and acceleration of the target center in the image plane, respectively. The measurement vector is expressed as $z_{k}={\left[x_{m,k},y_{m,k}\right]}^{T}$, representing the raw coordinates extracted from the morphological processing module.

The state transition equation and measurement equation are formulated as:

(1) $$x_{k}=Fx_{k-1}+w_{k-1},$$

(2) $$z_{k}=Hx_{k}+v_{k},$$

where $w_{k}∼N\left(0,Q\right)$ and $v_{k}∼N\left(0,R\right)$ represent the process noise and measurement noise vectors, respectively. At the *k*-th filter update, the sampling interval was calculated from the timestamps of two consecutive valid visual frames as $\Delta t_{k}=\tau_{k}-\tau_{k-1}$. The local visual-alignment stream operated at approximately 10–15 FPS; therefore, the typical inter-frame interval was approximately 0.067–0.100 s. The measured $\Delta t_{k}$, rather than a fixed nominal interval, was used to construct the state transition matrix for each valid update. Based on the kinematic rules with the sampling interval $\Delta t_{k}$, the state transition matrix *F* is structured as:

(3) $$F=\left[\begin{array}{cccccc} 1 & 0 & \Delta t & 0 & \frac{1}{2}\Delta t^{2} & 0\\ 0 & 1 & 0 & \Delta t & 0 & \frac{1}{2}\Delta t^{2}\\ 0 & 0 & 1 & 0 & \Delta t & 0\\ 0 & 0 & 0 & 1 & 0 & \Delta t\\ 0 & 0 & 0 & 0 & 1 & 0\\ 0 & 0 & 0 & 0 & 0 & 1 \end{array}\right].$$

The measurement matrix H, which maps the state space to the observation space, is given by:

(4) $$H=\left[\begin{array}{cccccc} 1 & 0 & 0 & 0 & 0 & 0\\ 0 & 1 & 0 & 0 & 0 & 0 \end{array}\right].$$

The Kalman parameters were selected through sequential empirical tuning during preliminary local visual–kinematic alignment trials conducted under the same camera configuration, low-speed chassis micro-adjustment condition, and mechanical-vibration environment as the reported experiments. The matrices *Q* and *R* were not estimated through an independent statistical noise-identification experiment; instead, they were treated as implementation-specific diagonal tuning parameters. During the preliminary trials, the values of *R* and *Q* were progressively adjusted. A candidate setting was retained only when it suppressed the visible high-frequency coordinate jitter induced by image-plane centroid fluctuations and mechanical vibration, did not introduce persistent lag during local alignment, and allowed the predefined convergence criterion of sub-pixel error for three consecutive valid frames to be reached reliably. The final measurement-noise covariance was set to $R=150\cdot I_{2}$, which reduces the sensitivity of the update to abrupt raw coordinate deviations, whereas the process-noise covariance was set to $Q=0.1\cdot I_{6}$ to preserve a smooth short-horizon prediction during low-speed micro-adjustment. The initial posterior error covariance was fixed at $P_{0}=0.1\cdot I_{6}$, and all three matrices were kept unchanged throughout the 100 independent placement trials reported in Section 4.6. The zero-mean Gaussian-white formulation for $v_{k}$ is used as a local filtering approximation for short-term image-plane centroid perturbations caused by vibration, segmentation fluctuation, and finite coordinate extraction; it does not assert that every disturbance source follows an exactly Gaussian distribution under all operating conditions. Likewise, the Constant Acceleration model is a short-horizon second-order predictor between consecutive valid visual frames, rather than an assumption that the chassis follows a prescribed constant-acceleration trajectory. The actual chassis motion remains determined by the current image-plane error through the X–Y PID controller. Figure 13 illustrates the resulting suppression of high-frequency coordinate fluctuations under the tested conditions.

## 4. Results

This section evaluates the main functional modules of the proposed IMR system, including visual obstacle avoidance, visual workpiece recognition, multi-sensor coordination, and visual localization. The results focus on detection performance, edge-deployment efficiency, sensor coordination, and positioning accuracy to verify the feasibility of the proposed system under the tested indoor industrial experimental conditions.

### 4.1. Verification of Visual Obstacle Avoidance

**Figure 14:**
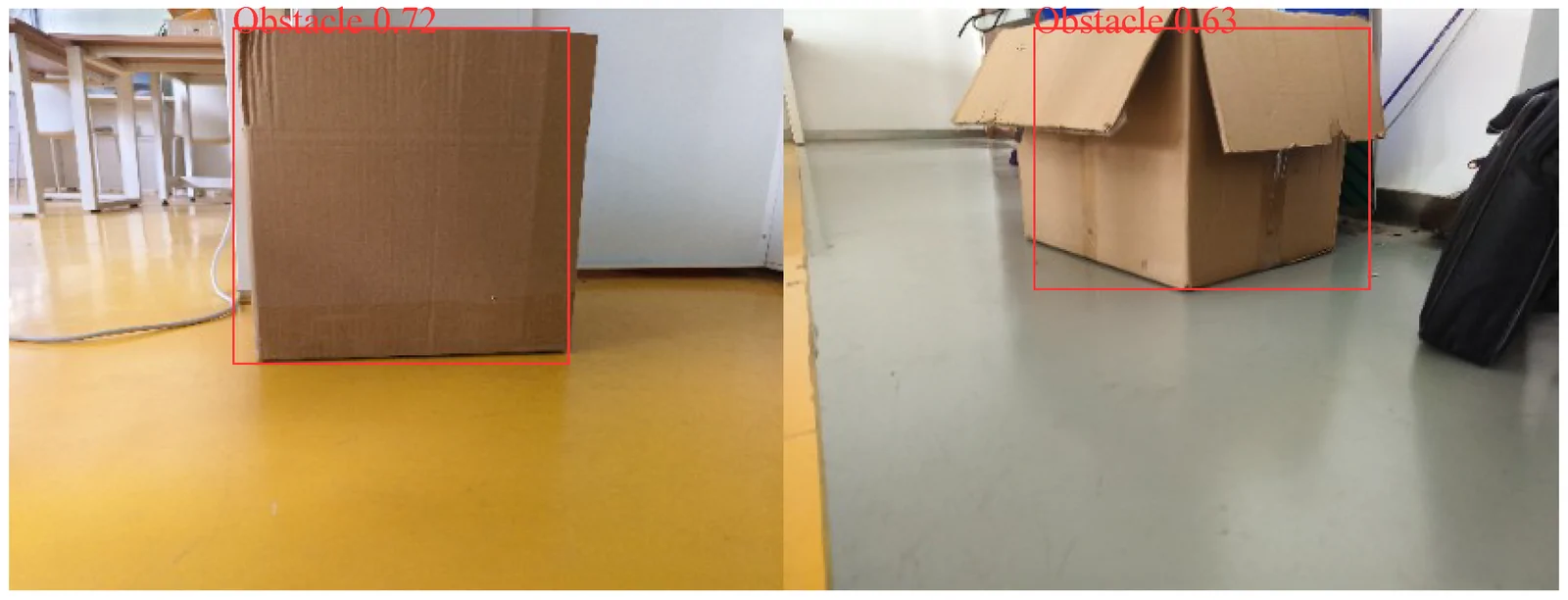
Real-time obstacle detection results during navigation.

Figure 14 shows representative real-time obstacle-detection frames from the navigation experiment. The visual module identifies obstacles in the camera view and provides their image-plane positions to the decision layer, where the predefined trigger zone generates the visual early-warning condition. These observations provide an early semantic cue; physical range and subsequent safety actions are determined by LiDAR and ultrasonic sensing as specified in Section 3. Figure 14 is a qualitative illustration of the single-class navigation-obstacle detector.

**Figure 15:**
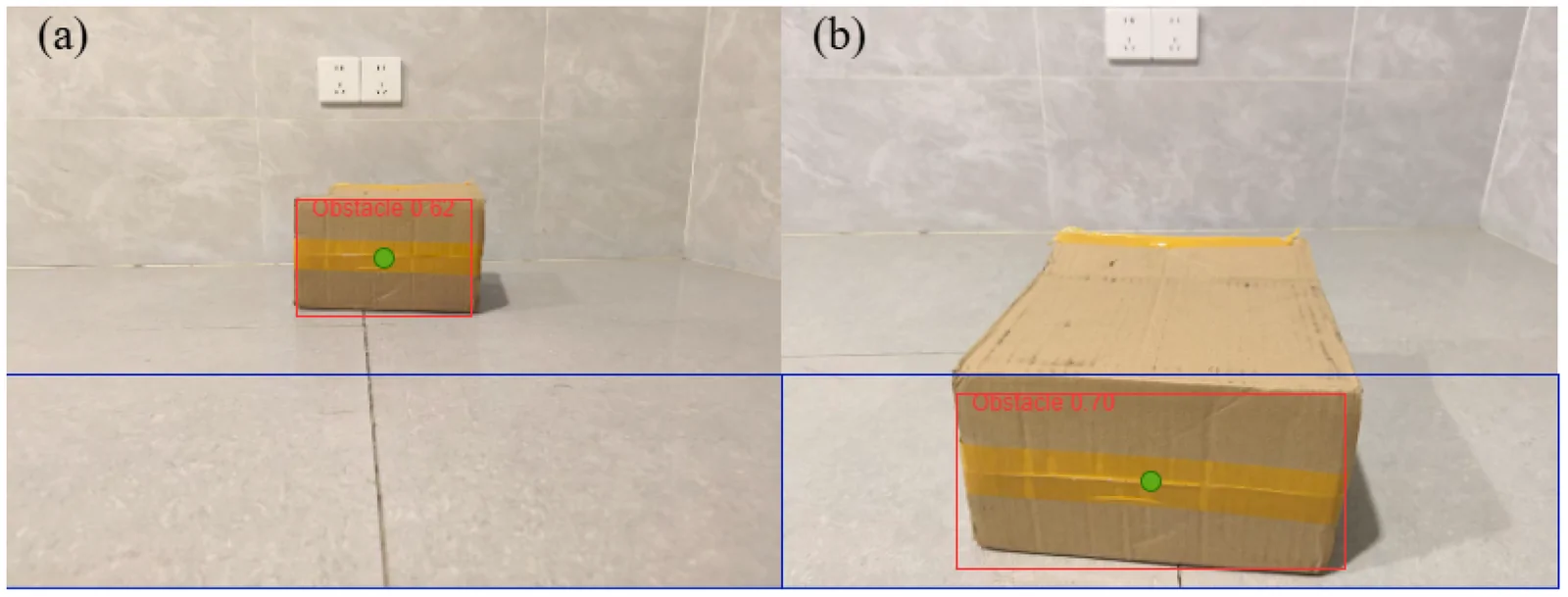
Visual obstacle-trigger logic: (**a**) obstacle center outside the image-plane trigger zone; (**b**) obstacle center entering the image-plane trigger zone.

As shown in Figure 15, the visual module detects the obstacle and calculates its image-plane center point, represented by a green dot. In Figure 15a, the center point is outside the pre-defined blue trigger zone, whereas in Figure 15b, it enters this zone. The trigger zone is specified in image coordinates and is used solely to generate a two-dimensional visual obstacle-trigger event; it does not estimate the physical distance between the IMR and the obstacle. Physical range information and the associated safety constraints are independently provided by the LiDAR and ultrasonic sensors. The fused decision layer combines the visual trigger information with these range measurements to determine the subsequent motion-control action.

**Figure 16:**
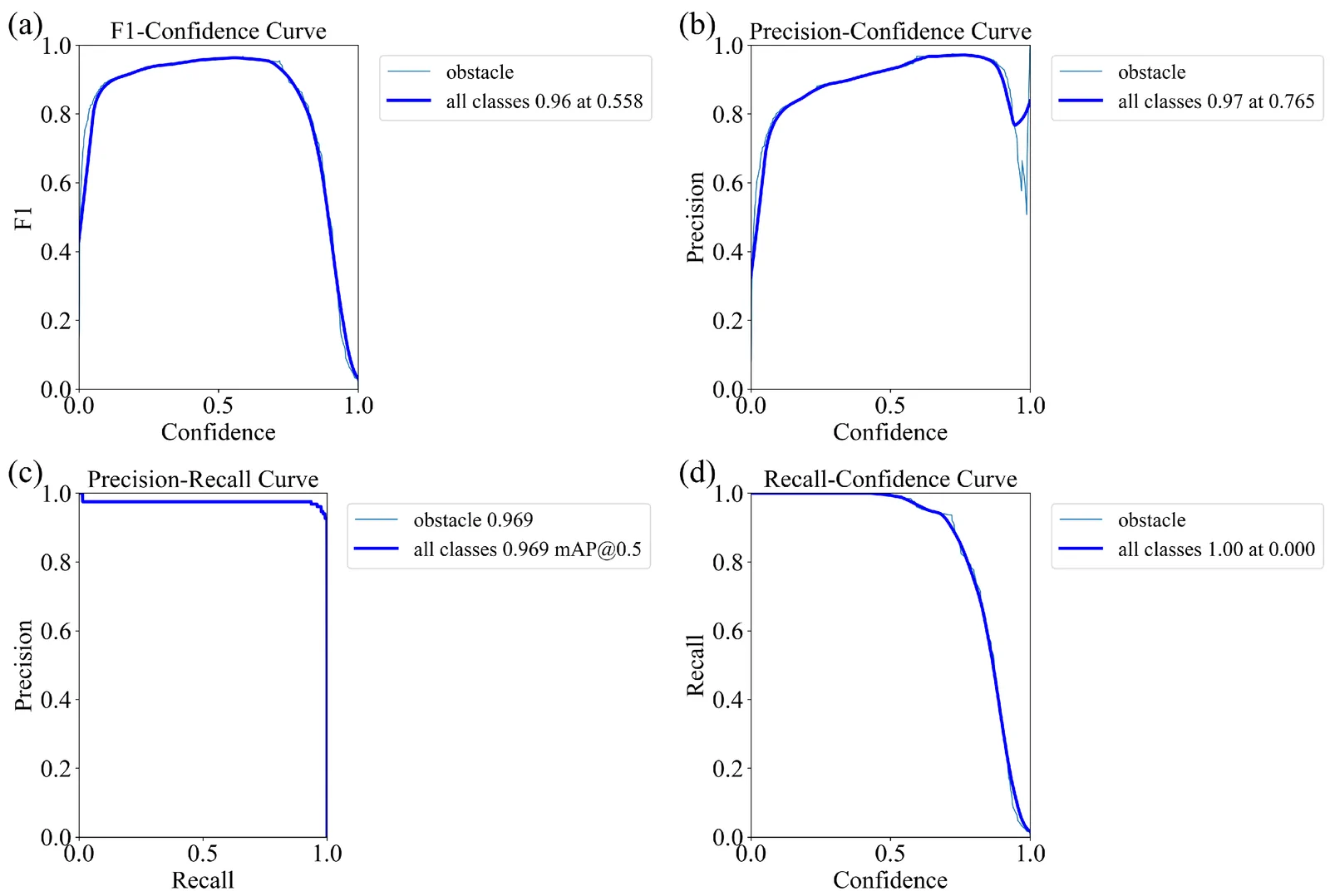
Quantitative performance of the single-class obstacle detector: (**a**) F1–confidence curve; (**b**) Precision–confidence curve; (**c**) Precision–Recall curve; and (**d**) Recall–confidence curve.

To quantitatively evaluate the single-class obstacle-detection task, the Precision–confidence, Recall–confidence, F1–confidence, and Precision–Recall curves are presented in Figure 16. The all-class Precision–confidence curve reaches a maximum precision of 0.96 at a confidence threshold of 0.754, whereas the Recall–confidence curve reaches a maximum recall of 1.00 at a threshold of 0.000. The F1–confidence curve attains a maximum F1-score of 0.96 at a confidence threshold of 0.558. The corresponding Precision–Recall curve yields an mAP@0.5 of 0.961 for the obstacle category. Because the maximum precision, recall, and F1-score occur at different confidence thresholds, these values characterize the threshold-dependent behavior of the detector rather than a single operating-point result.

### 4.2. Performance Verification of Visual Workpiece Recognition

#### Dataset Construction

The experiments were conducted on a system running Windows 11 Pro (24H2), powered by a 13th Gen Intel(R) Core(TM) i5-13500H processor (2.60 GHz). The experimental setup also included an NVIDIA GeForce RTX 4060 Ti graphics card with 8 GB of VRAM and 16 GB of system memory. The software environment consisted of Python 3.10 and PyTorch 1.10.2.

Due to the lack of publicly available datasets for workpiece detection in machining workshops, a custom dataset was independently collected and manually annotated for this experiment. The dataset contains 1720 images of four common workpiece categories: bolt, nut, screw, and washer. The images were acquired in an indoor industrial experimental environment that simulated practical workpiece-recognition conditions during mobile-platform or manipulator operations. A MaixCAM camera was mounted at the end of the manipulator and principally oriented vertically downward. Camera-to-target distances ranged from 0.2 m to 1.5 m, while small pose variations within 0–20° were included to represent practical placement variations. To construct samples with different spatial distributions, the positions, quantities, and mutual occlusion relationships of the workpieces within the working area were varied, including single-target, multiple-target, and partially overlapping configurations. The images covered normal overhead illumination, local shadow regions, and different background-reflection conditions. The background areas included workbench surfaces, surrounding equipment areas, and placement regions with different colors and textures.

The dataset was divided into 1380 training images, 170 validation images, and 170 test images. All images were manually annotated using LabelImg with rectangular bounding boxes for the bolt, nut, screw, and washer categories. Following the initial annotation, all labels were manually reviewed for category consistency, bounding-box completeness, and bounding-box location accuracy. Samples with ambiguous boundaries, target occlusion, or uncertain category assignments were re-annotated or excluded before finalizing the dataset. During training, the input resolution was set to 448×448, and the model was trained for 200 epochs with a batch size of 16.

**Figure 17:**
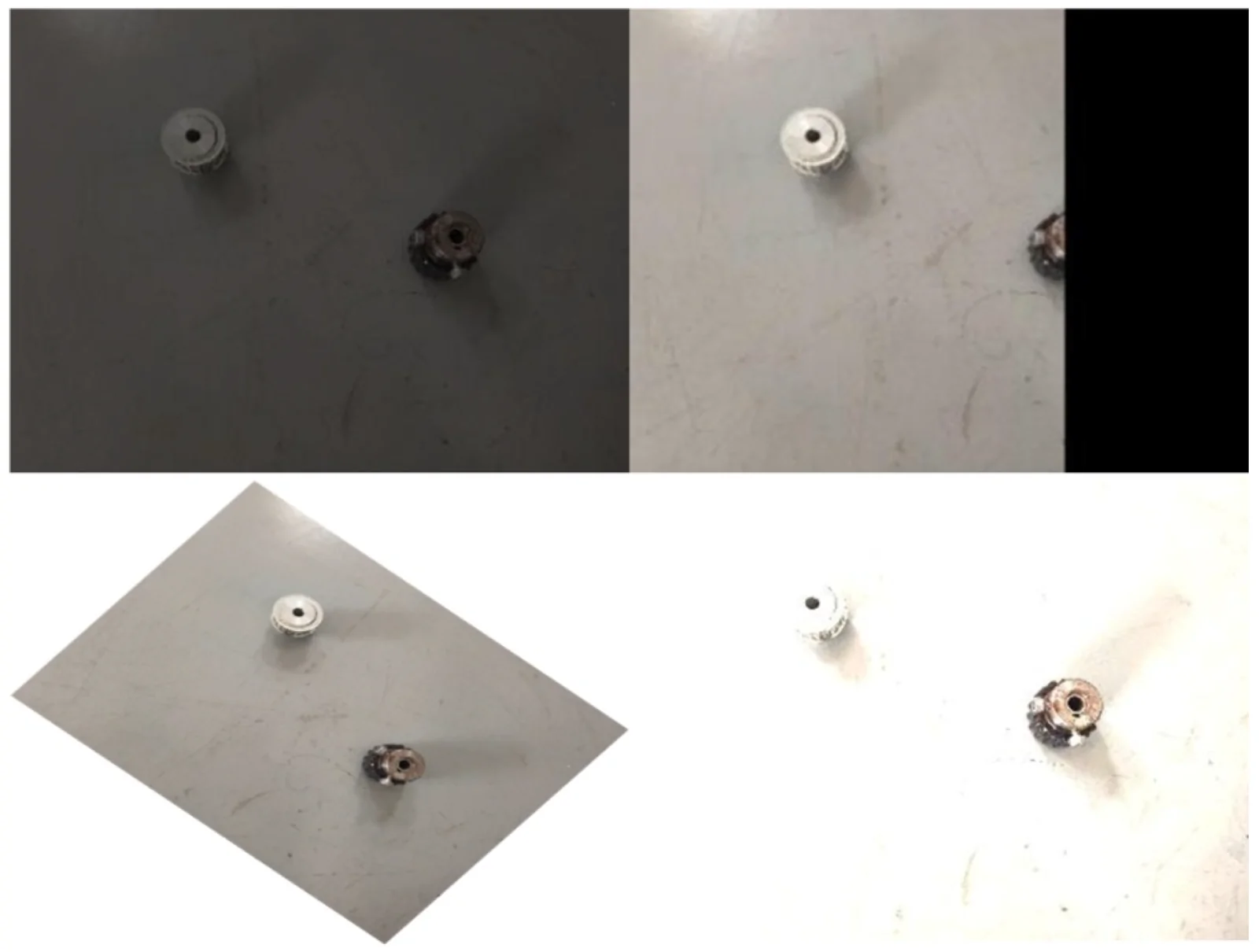
Construction and augmentation of the dataset.

As shown in Figure 17, to better simulate the effects of lighting and viewing-angle variations in the machining workshop on the recognition performance of the YOLO model, several data augmentation techniques were applied to the training set. The original images underwent random blurring, random rotation, and brightness adjustment (lowering and raising). To enhance YOLOv5s’s capability to recognize occluded objects in the workshop, target truncation and random “cutout” (using black blocks to simulate occlusion) were employed. Furthermore, rotation was used to simulate changes in workpiece placement angles, while brightness variations simulated scenarios such as workpiece reflection and local overexposure caused by direct workshop lighting.

**Figure 18:**
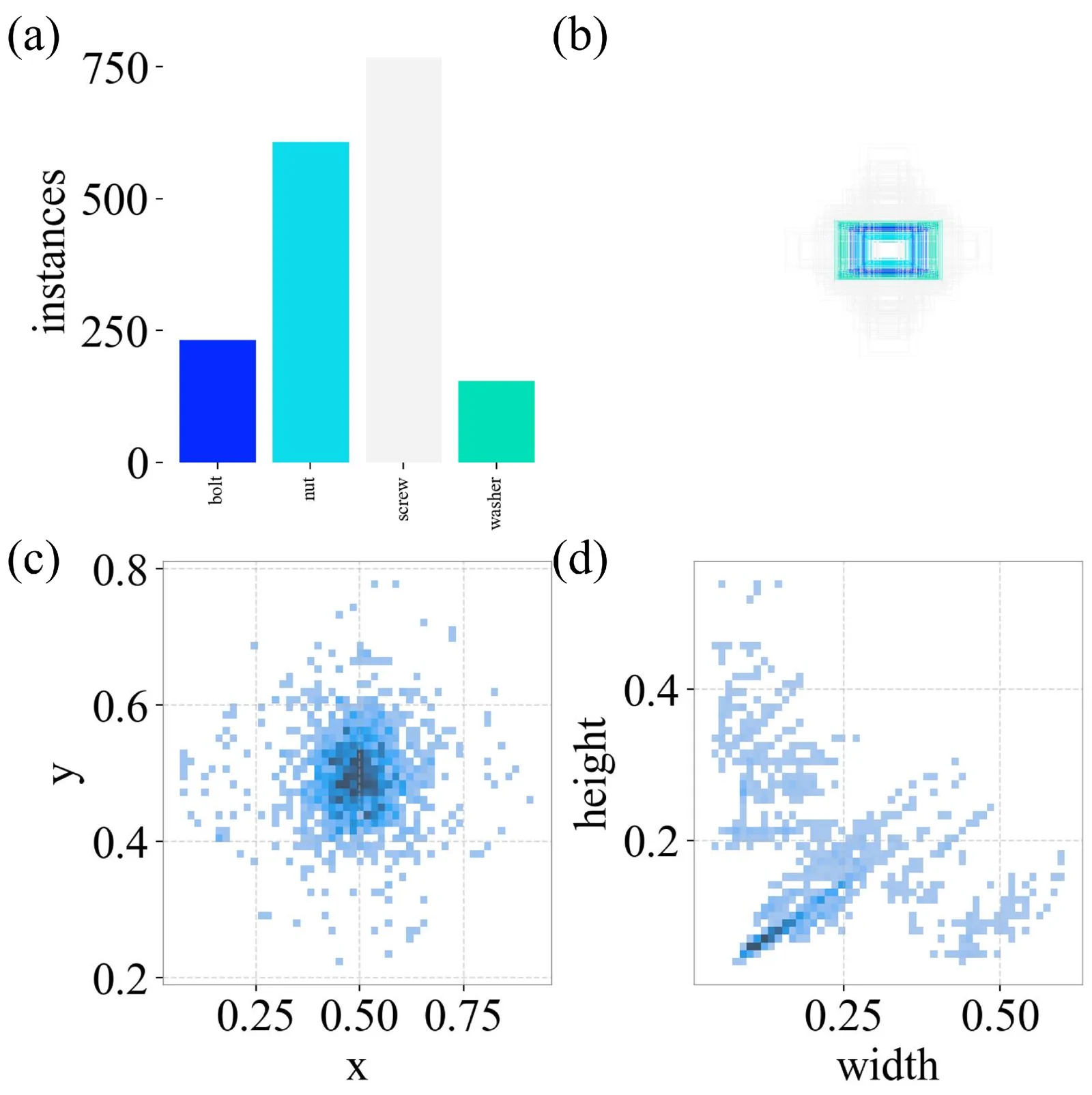
Statistical analysis of the training dataset: (**a**) class distribution; (**b**) bounding box dimensions; (**c**) center coordinate distribution; (**d**) target scale correlation.

To verify the quality of the dataset and optimize the training strategy of the network model, the label information of the training set was analyzed, including the class distribution, bounding-box dimensions, center-coordinate distribution, and target-scale correlation, as shown in Figure 18.

**Figure 19:**
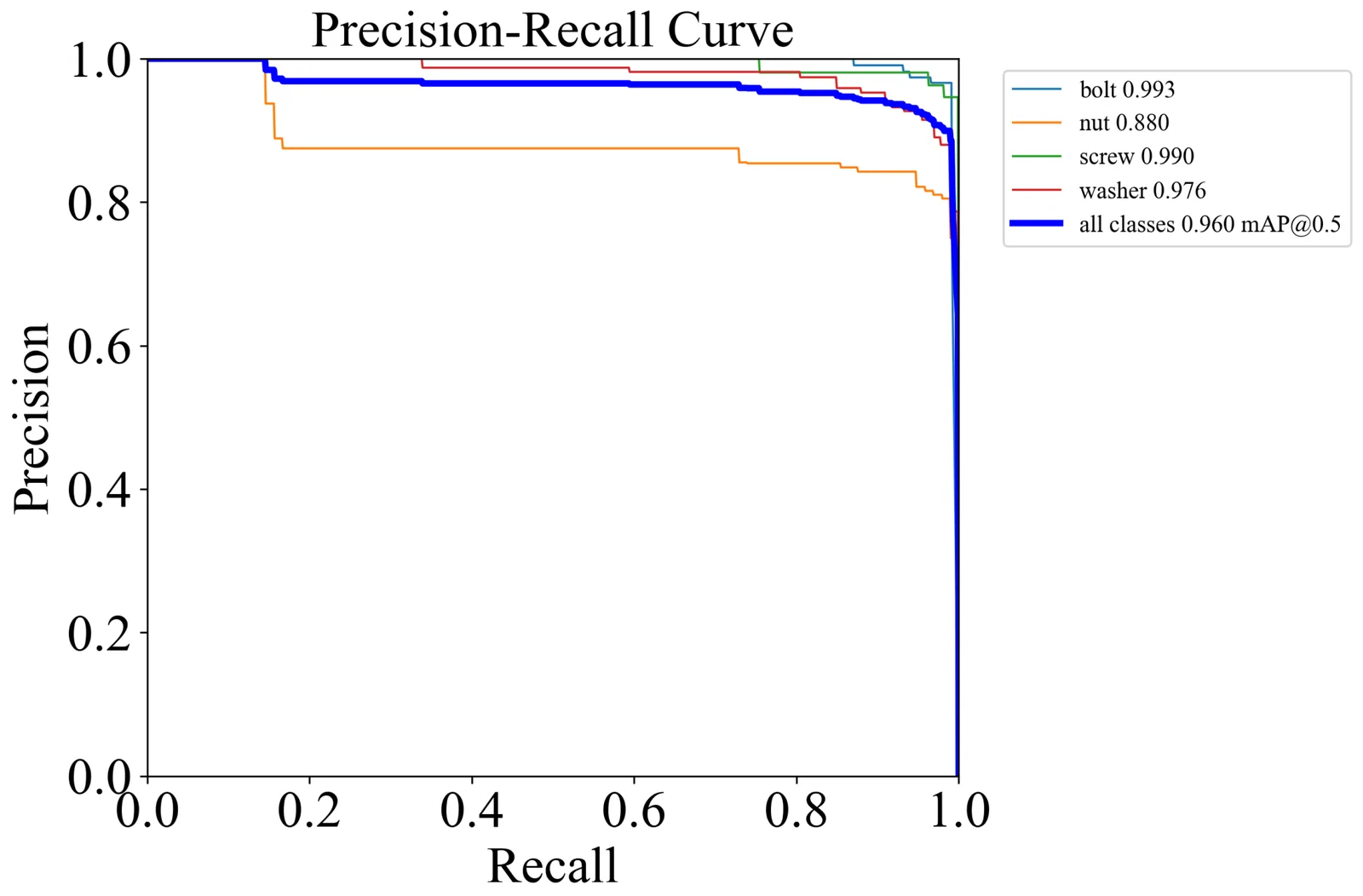
Class-wise Precision–Recall curves of YOLOv5s for bolt, nut, screw, and washer recognition.

- **Class Distribution:** As shown in the top-left chart, the dataset contains four workpiece categories: bolt, nut, screw, and washer. The instance counts differ across categories, with nut and screw instances occurring more frequently than bolt and washer instances. Therefore, the class-wise Precision–Recall curves in Figure 19 are reported to assess the recognition performance of each category separately.
- **Target Size and Shape:** The top-right and bottom-right charts show the distributions of normalized bounding-box width, height, and target-scale correlation. The workpieces exhibit different size and shape characteristics, reflecting the geometric differences among bolts, nuts, screws, and washers. These variations provide training samples with multiple target scales and aspect profiles.
- **Center Point Distribution:** The bottom-left chart shows that most targets are concentrated around the central region of the image coordinate system, while some targets occur at off-center positions. To increase the positional diversity of training samples, Mosaic data augmentation was adopted by randomly stitching images together, thereby increasing the occurrence of targets in edge regions.

To characterize the offline detection capability of the trained FP32 model before edge-model conversion, the model was evaluated on a desktop GPU using the held-out validation set and standard object-detection metrics, including Precision, Recall, F1-score, and mean Average Precision (mAP) [34,35]. Figure 19 presents the class-wise Precision–Recall curves for the four workpiece categories. The AP@0.5 values for bolt, nut, screw, and washer were 0.993, 0.880, 0.990, and 0.976, respectively, and the overall offline-validation mAP@0.5 was 0.960. The Precision, Recall, and F1-score were additionally evaluated across the confidence-threshold sweep. Their all-class peak values were 1.00, 0.98, and 0.80, respectively; however, these extrema occurred at different confidence thresholds and should therefore not be interpreted as a single operating-point result. Among the four categories, nut achieved the lowest AP@0.5, while the confusion matrices in Figure 7 and Figure 8 further show that nut–washer discrimination remains the principal category-dependent error mode. Thus, the results support the use of the model for four-category workpiece detection under the tested imaging conditions, while indicating that recognition of visually similar circular workpieces requires further improvement.

Figure 7 and Figure 8 evaluate the pruned INT8 model deployed on MaixCAM, whereas Figure 19 reports FP32 offline validation before model conversion. These results are not directly comparable: the deployment confusion matrices describe category-assignment errors of the converted model, while the offline mAP@0.5 summarizes the precision–recall behavior of the trained FP32 model. The nut–washer confusion observed in the deployment results is therefore interpreted as a category-specific limitation of the current edge configuration under the tested conditions.

**Figure 20:**
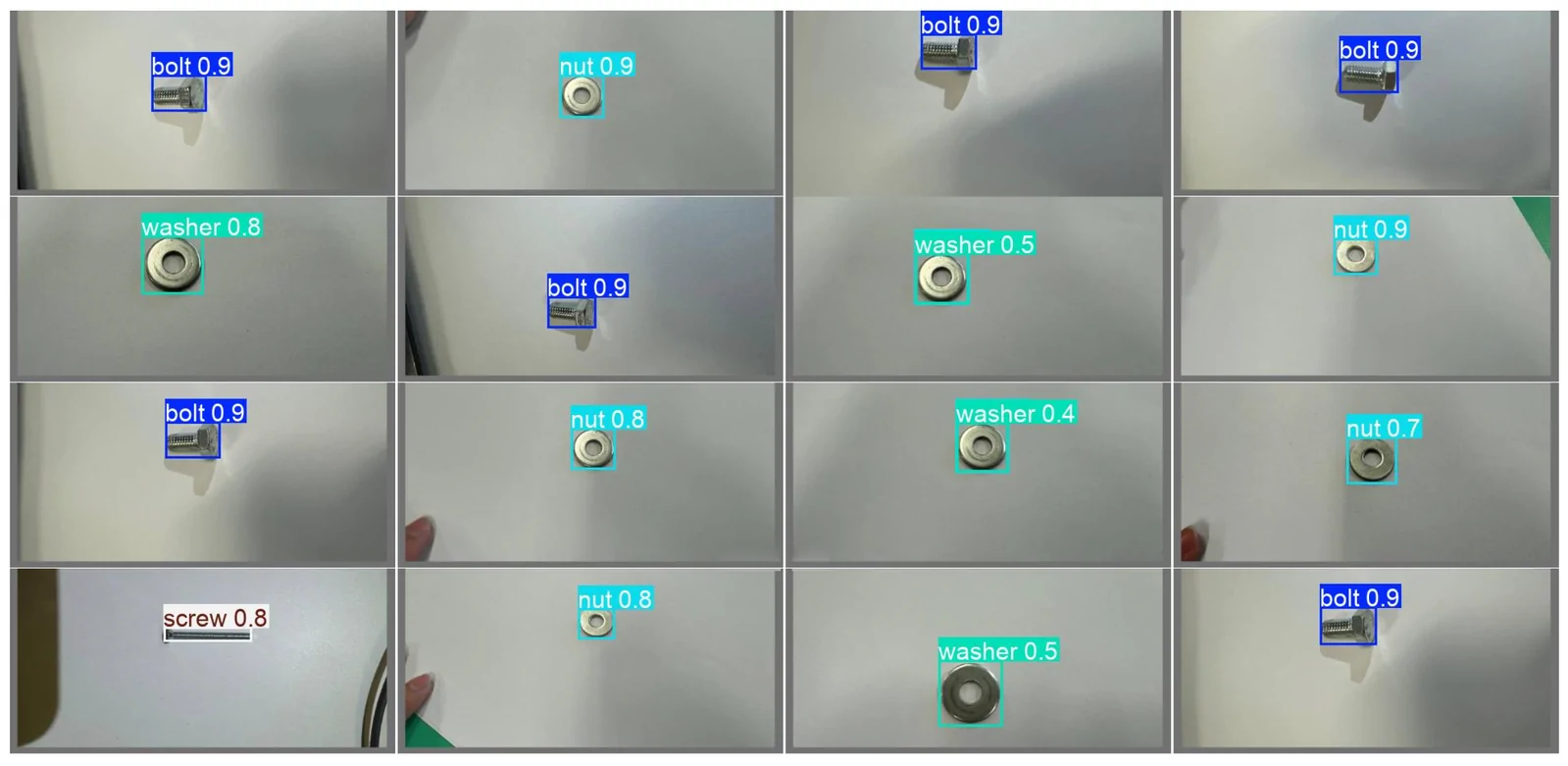
Representative detection results for four workpiece categories: bolt, nut, screw, and washer.

To qualitatively assess the trained YOLOv5s model on representative validation images, four workpiece categories—bolt, nut, screw, and washer—were processed, as shown in Figure 20. The predicted bounding boxes localize the detected workpieces and are annotated with the corresponding predicted category and confidence score. These representative results complement the quantitative Precision–Recall evaluation in Figure 19 and demonstrate the model’s ability to distinguish the four workpiece categories under the tested imaging conditions.

Given the limited computational resources of the MaixCAM platform, which exclusively supports INT8 integer arithmetic and 3×3 convolutions, model pruning and quantization were implemented to facilitate the deployment of YOLOv5s. We adopted a pruning strategy based on the scaling factor of the batch normalization (BN) layer. In the YOLOv5s architecture, convolutional layers are followed by BN layers, each containing a trainable scaling factor, γ. During training, an L1 regularization penalty term targeting this γ value was incorporated into the loss function, forcing the γ values of a subset of channels to approach zero. Consequently, channels with γ values nearing zero and their corresponding convolutional kernels were directly pruned. By eliminating 36% of the low-contribution channels within the convolutional layers, both the parameter count and computational load were significantly reduced.

Following pruning, the INT8 quantization method was employed. Its core principle involves mapping floating-point values to a low-bit integer domain, as described by the following equation:

(5) $$Q\left(x\right)=\mathrm{round}\left(\frac{x}{scale}\right)+zero\_point,$$

where scale and zero_point are the quantization parameters. To determine the optimal scale and mitigate excessive accuracy loss, a calibration dataset was utilized. The Kullback–Leibler (KL) divergence algorithm was applied to minimize the information error between the distributions before and after quantization. The principle is defined as follows, where *P* is the original distribution and *Q* is the quantized distribution:

(6) $$D_{KL}\left(P\|Q\right)=\sum P\left(x\right)\log\frac{P(x)}{Q(x)}.$$

Post-quantization, the model volume was reduced to 34% of its original size. On the Jetson Nano benchmark platform, latency decreased from 91.5 ms to 27.8 ms (a 69.6% reduction), with an accuracy loss of less than 2% mAP.

Finally, since the MaixCAM platform requires models in the .mud format, a specific conversion pipeline was executed. First, the trained .pt model was exported to an .onnx model with an input resolution of 448×448 using a YOLOv5s-compatible PyTorch framework. Utilizing the TPU-MLIR toolchain within a Docker environment, the .onnx model was subsequently converted into a .cvimodel. This process involved configuring the model input shape as {1,3,448,448}, applying preprocessing parameters (*mean* and *scale*) consistent with the .pt training, and enabling INT8 quantization. The resulting .cvimodel was then configured via a .mud file and deployed to the MaixCAM, thereby achieving lightweight model deployment.

### 4.3. Comparative Analysis of Edge Model Inference Performance

This section compares the edge-deployment performance of YOLOv5s, YOLOv8s, and YOLOv11s under the same MaixCAM hardware and deployment conditions. The comparison considers deployment accuracy, inference speed, latency, and runtime memory consumption to assess model suitability for the resource-constrained edge platform.

#### 4.3.1. Performance Benchmark of YOLOv5s, YOLOv8s, and YOLOv11s

Under the same MaixCAM hardware, 448 × 448 input resolution, and INT8 deployment setting, YOLOv5s achieved a deployment mAP@0.5 of 62.5% and an inference speed of 24.5 FPS, whereas YOLOv8s achieved 63.8% mAP@0.5 at 13.2 FPS. Latency was measured by inserting temporal hooks into the Python deployment pipeline and was decomposed into image capture, pre-processing, inference, and post-processing stages. The corresponding total pipeline latencies were 40.8 ms for YOLOv5s and 75.7 ms for YOLOv8s. YOLOv5s therefore exceeded the predefined real-time criterion of 20 FPS, whereas YOLOv8s did not.

The main performance difference occurred in the inference and post-processing stages. YOLOv5s required 21.5 ms for deployment inference and 12.1 ms for non-maximum suppression (NMS), resulting in a total latency of 40.8 ms. In comparison, YOLOv8s required 44.8 ms for inference and 23.7 ms for post-processing. The heavier computational and decoding workload of YOLOv8s therefore resulted in substantially higher latency on the tested embedded platform.

Although YOLOv8s provided a 1.3-percentage-point improvement in mAP@0.5 over YOLOv5s, this gain was accompanied by a substantial reduction in inference speed and an increase in runtime memory consumption. YOLOv8s operated at 13.2 FPS and required 168 MB of runtime memory, compared with 24.5 FPS and 116 MB for YOLOv5s. Therefore, under the tested MaixCAM constraints, the modest accuracy improvement of YOLOv8s did not compensate for its loss in real-time performance and memory margin. The detailed edge-inference results are summarized in Table 1.

**Figure 21:**
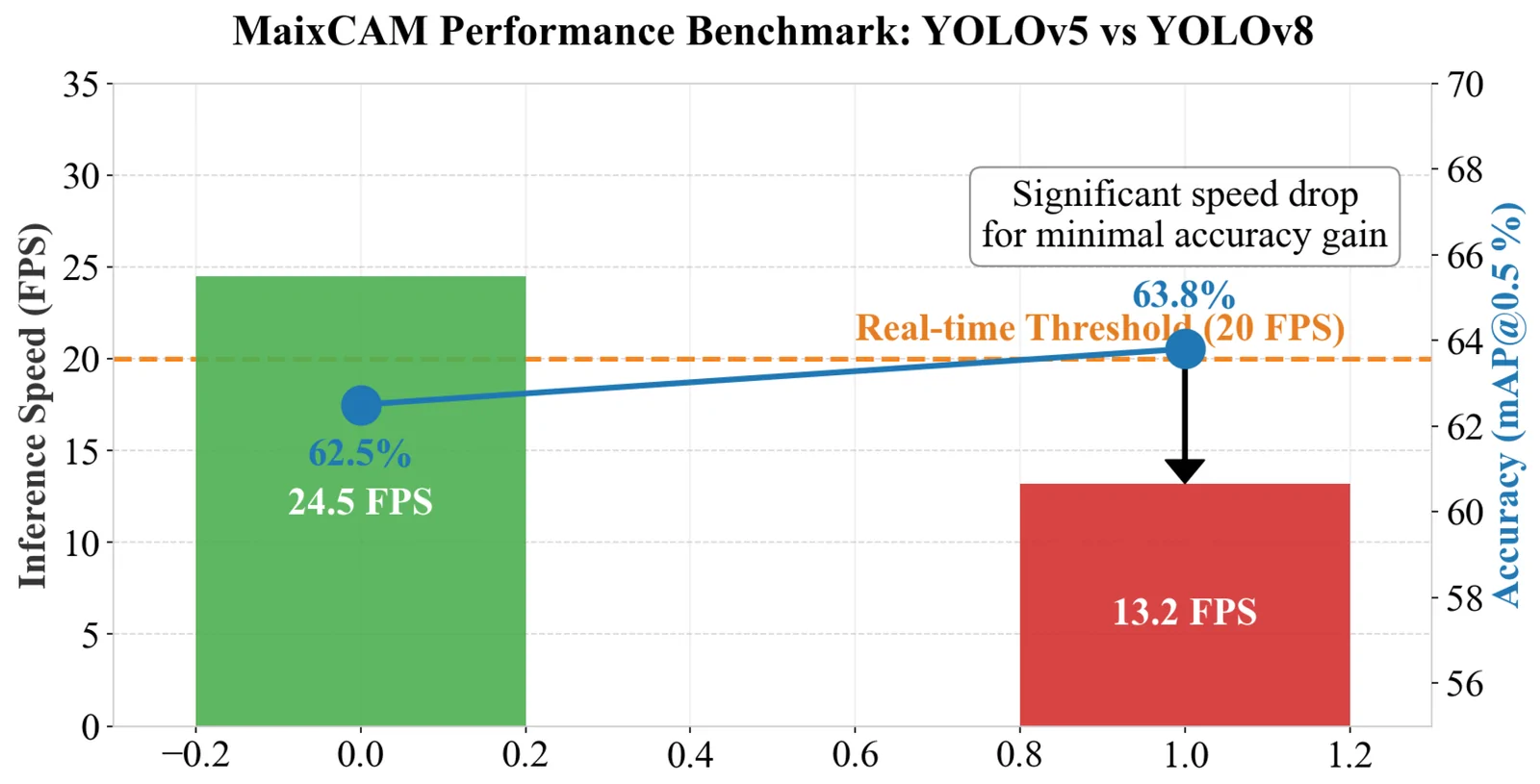
Device-side inference performance comparison of YOLOv5s and YOLOv8s on the MaixCAM platform.

YOLOv11s was further examined under the same MaixCAM hardware, 448 × 448 input resolution, and INT8 deployment pipeline. However, the model could not complete the required conversion and deployment procedure because its runtime memory requirement exceeded the available 256 MB memory of the platform. Consequently, no valid edge-side mAP@0.5, inference-speed, latency, or runtime-memory values are reported for YOLOv11s. This result represents a deployment-feasibility limitation rather than a comparison of its detection accuracy with the other models. In the proposed system, YOLOv5s serves as an auxiliary semantic-perception module that supplies obstacle or workpiece category information to the decision-level coordination mechanism. The model selection was therefore based not on the highest detection accuracy alone but on the joint requirements of deployment compatibility, real-time response, runtime memory, and sufficient recognition performance. Under these conditions, YOLOv5s provided the most suitable accuracy–speed–memory trade-off for the implemented MaixCAM–STM32 system. The device-side inference performance comparison of YOLOv5s and YOLOv8s is shown in Figure 21.

#### 4.3.2. Comparison of Baseline and Edge-Optimized YOLOv5s Deployment Models

To quantify the deployment effect of the proposed edge-optimization procedure, the baseline YOLOv5s deployment model was compared with the edge-optimized deployment model under the same experimental configuration. The procedure combines structural channel pruning, detection-head adaptation, and INT8 quantization for MaixCAM deployment. All experiments were conducted using the same dataset, input resolution (448 × 448), hardware platform, and deployment environment to ensure a fair comparison.

As summarized in Table 2, the baseline YOLOv5s deployment model contains 7.02 M parameters and occupies 14.1 MB of model storage. After structural channel pruning, detection-head adaptation, and INT8 quantization, the edge-optimized deployment model contains 4.49 M parameters and occupies 4.8 MB. Its inference speed increases from 11.2 FPS to 24.5 FPS, while runtime memory consumption decreases from 182 MB to 116 MB. The corresponding deployment mAP@0.5 decreases from 64.1% to 62.5% after model compression. Thus, the measured results characterize an edge-deployment trade-off: the optimized model sacrifices a limited amount of deployment mAP@0.5 to satisfy the real-time and memory constraints of the MaixCAM platform.

### 4.4. Hardware-Level Emergency Braking Metrology and Intrusion Testing

To rigorously quantify the hardware-level emergency response capability against perceptual blind spots, a standardized transparent acrylic panel intrusion test was designed. The experiment was repeated 20 times under a constant initial chassis velocity of 0.5 m/s (deliberately configured to exceed the maximum normal operational speed of 0.4 m/s to conduct a rigorous over-speed stress test). A highly transparent acrylic board was abruptly introduced directly in front of the vehicle, intentionally breaking the 0.3 m ultrasonic absolute safety threshold.

To ensure the validity of the reported metrics, the measurement metrology was explicitly defined: (1) **Timing Start Point ($t_{0}$)**: Registered by the STM32 internal hardware timer (operating at a 1 kHz sampling frequency) at the exact millisecond the ultrasonic echo breached the 0.3 m logic threshold, triggering the 3.3 V interrupt signal. The motor-command timestamp ($t_{\mathrm{cmd}}$) was recorded by the same timer when the hardware-interrupt service routine issued the reverse-braking/full-stop PWM command to the four Mecanum motor drivers. (2) **Velocity Acquisition**: Chassis velocity was continuously monitored via the high-resolution Hall-effect encoders mounted on the four Mecanum wheels, sampled at 100 Hz within the PID control loop. The final encoder edge was time-stamped using timer input capture, and a zero-velocity state was confirmed after a predefined no-pulse confirmation interval. (3) **Timing Endpoint ($t_{1}$)**: Defined as the moment the encoder readings consistently reached and maintained absolute zero velocity. The overall response time ($\Delta t=t_{1}-t_{0}$) encompasses the sensor trigger delay, MCU interrupt processing overhead, and the electro-mechanical braking execution.

**Figure 22:**
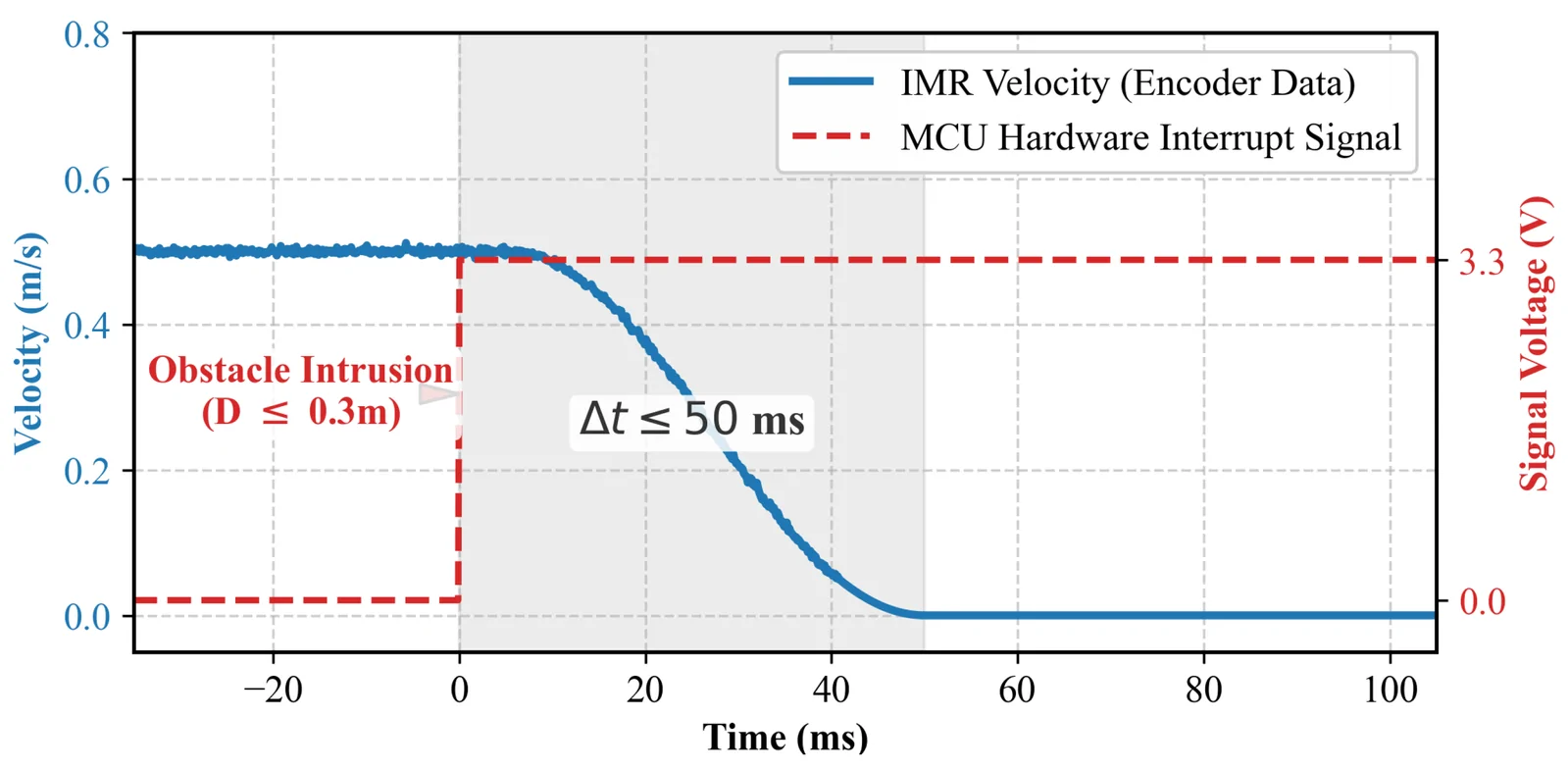
Dynamic braking response showing low-latency emergency stopping triggered by the MCU hardware interrupt (data from a representative trial).

Figure 22 illustrates the dynamic response curve of a representative trial. As illustrated in Figure 22, the ultrasonic array bypassed the standard high-latency ROS communication overhead upon detecting the intrusion, triggering a direct MCU hardware interrupt (3.3 V) to minimize response latency. Statistical analysis across all 20 repeated trials indicates an average response time of 48.2±2.4 ms and an average physical braking distance of 1.42±0.08 cm. The trigger-to-command delay and command-to-zero-velocity braking duration were 8.5±0.8 ms and 39.7±2.1 ms, respectively. At the declared stopping endpoint, the residual velocity was 0.00 m/s in all trials. The final physical clearance between the front rigid reference point of the IMR and the acrylic panel, measured after complete stopping using a steel ruler, was 28.6±0.1 cm, with a worst-case clearance of 28.4 cm. The detailed braking statistics are summarized in Table 3. It must be noted that while these metrics represent the deterministic physical lower bound of the chassis under a specific controlled straight-line intrusion condition and should not be misconstrued as a universal performance guarantee for all dynamic scenarios, they support the low-latency advantage of the hardware interrupt mechanism compared to standard software-level obstacle avoidance.

### 4.5. System-Level Quantitative Evaluation Under Multi-Variable Industrial Scenarios

#### 4.5.1. System-Level Comparison of Functional Configurations

To systematically evaluate the robustness and efficiency of the proposed decision-level multi-sensor coordination strategy, comprehensive navigation experiments were conducted under a highly randomized multi-variable matrix. The testing pipeline was deployed across two distinct manufacturing layouts: Layout A (characterized by narrow, structured factory aisles) and Layout B (characterized by unstructured, open material transfer zones). Within these environments, three dynamic pedestrian and obstacle densities were established: Low ($0.05\;{\mathrm{obs}/m}^{2}$), Medium ($0.15\;{\mathrm{obs}/m}^{2}$), and High ($0.30\;{\mathrm{obs}/m}^{2}$). To fully replicate workshop disturbances, the ambient illumination dynamically fluctuated between 200 and 1000 lux, while the robot operational velocity was varied from 0.2m/s to 0.4m/s to match typical industrial intralogistics pacing.

A total of 50 randomized trials were performed for each of the three functional configurations: LiDAR-only navigation, standard Vision+LiDAR fusion (without hierarchical interrupt control), and the proposed Full System. Table 4 summarizes the quantitative performance metrics across these multi-variable stress tests.

The LiDAR-only configuration achieved a success rate of only 62%, suffering 19 rigid collisions primarily due to perceptual blind spots caused by transparent materials. The standard Vision+LiDAR fusion configuration leveraged semantic awareness for pedestrians to improve the success rate to 82%; however, it still recorded 9 collisions. This failure stems fundamentally from the optical properties of transparent materials. Specifically, the high light transmittance and specular reflection under fluctuating factory illumination (200–1000 lux) severely hindered YOLOv5s from extracting effective features. Consequently, the pure vision system remained inherently blind to near-field acrylic intrusions, leading to collisions regardless of the operational speed.

In contrast, the proposed full hierarchical system introducing the ultrasonic-triggered hardware interrupt recorded no rigid collisions across all tested layouts, lighting variations, and chassis speeds. The proposed system maintained a 96% absolute navigation success rate. In the remaining 4% of extreme corner cases—specifically defined by dense spatial constraints and simultaneous transparent obstacle intrusions—the low-level hardware interrupt safely braked the robot to a complete stop, though these complex constraints induced local costmap deadlocks that required manual recovery. Accounting for the relative approach momentum of dynamic obstacles during the Δt≤50ms braking window, the minimum dynamic safety distance was reliably bounded at 0.18 m. These statistical results indicate that, under the tested layouts, obstacle densities, illumination range, and velocity settings, the hierarchical architecture improved the safety margin while maintaining competitive task efficiency.

Table 4 provides a system-level ablation of the proposed hierarchical decision-level safety policy using five complementary performance metrics. The three configurations are explicitly defined as follows: LiDAR-only activates LiDAR-based geometric navigation, local-costmap updating, and local replanning; Vision + LiDAR additionally activates the vision-based semantic/image-plane trigger for preemptive deceleration; and the Full System additionally activates the ultrasonic hardware-interrupt safeguard. Thus, the comparison progressively evaluates the complementary decision functions of LiDAR, vision, and ultrasonic sensing under the same tested layouts, obstacle densities, illumination conditions, and chassis velocities.

The results quantify the contribution of each decision layer using five complementary metrics. LiDAR-only navigation achieved a 62% success rate, 19 rigid collisions, a minimum safety distance of 0.00 m, a completion time of 48.5 s, and a recovery time of 9.2 s. Adding the vision-triggered preemptive deceleration layer increased the success rate to 82%, reduced collisions to 9, and improved completion and recovery times to 39.8 s and 6.8 s, respectively. Adding the ultrasonic hardware-interrupt layer increased the success rate to 96%, recorded no rigid collisions in the 50 trials conducted under the tested conditions, yielded a minimum recorded safety distance of 0.18 m, and reduced completion and recovery times to 34.2 s and 1.8 s, respectively. These controlled comparisons support the separate and complementary roles of LiDAR geometric replanning, vision-based semantic early warning, and ultrasonic near-field safety override within the proposed decision-level multi-sensor coordination policy.

#### 4.5.2. Threshold-Sensitivity Evaluation

To examine the sensitivity of the decision policy to the two action-priority boundaries, one-factor threshold experiments were conducted using the proposed full system. For each tested setting, the non-tested threshold was fixed at its baseline value, and all sensing modalities remained active throughout the trial. Each setting was evaluated in 50 randomized navigation trials under the same two layouts, obstacle-density levels, illumination range of 200–1000 lux, and chassis velocity range of 0.2–0.4 m/s described above. The trial outcomes were mutually exclusive and were classified as autonomous success, rigid collision, or local-costmap deadlock/manual-recovery requirement. The completion time was calculated only for trials that autonomously reached the goal without collision or manual recovery. The minimum noncontact clearance was calculated over the trials without physical contact; consequently, this metric is reported separately from the rigid-collision rate.

As shown in Table 5, reducing $D_{\mathrm{ultra}}$ from 0.3 m to 0.2 m reduced the autonomous-success rate from 96% to 84% and produced an associated 16% rigid-collision rate, indicating that the reduced near-field margin was insufficient for a subset of dynamic intrusion conditions. Increasing $D_{\mathrm{ultra}}$ to 0.4 m eliminated collisions but did not improve the autonomous-success rate beyond the baseline; instead, more conservative emergency stopping increased the mean completion time from 34.2 s to 41.5 s. For the vision-related upper decision boundary, setting $D_{\mathrm{vis}}$ to 1.0 m delayed preemptive deceleration and resulted in an 80% autonomous-success rate, including 6% rigid collisions and 14% deadlock/manual-recovery cases. Increasing $D_{\mathrm{vis}}$ to 2.0 m improved the autonomous-success rate to 98% and increased the minimum noncontact clearance to 0.21 m, but prolonged the mean completion time to 43.6 s because deceleration was initiated earlier than necessary in many traversable situations. Therefore, the baseline pair of $D_{\mathrm{ultra}}=0.3$ m and $D_{\mathrm{vis}}=1.5$ m was retained as a practical operating point that maintained zero rigid collisions, a 0.18 m minimum noncontact clearance, and a comparatively short autonomous completion time under the tested conditions.

### 4.6. Performance Verification of High-Precision Task-Space Planar Positioning

The visual region localization module provides the visual feedback required for high-precision task-space planar positioning in the IMR’s precision grasping and placement tasks. It combines HSV color-space conversion, morphological operations, and Kalman filtering to estimate and smooth the image-plane center of the positioning marker. The planar positioning results reported in this section are independent of the four-class workpiece-recognition results and the labels correlogram in Figure 9. The local alignment pipeline uses HSV segmentation, morphological restoration, and six-dimensional constant-acceleration Kalman filtering to estimate the image-plane center of the positioning marker, and the resulting coordinate offset is used for closed-loop X–Y adjustment of the mobile base. During the placement stage, the relative geometry between the manipulator and the mobile base is fixed, the end-effector orientation is constrained by the mechanical structure, and the Z-axis placement stroke terminates at a fixed physical limit. Therefore, the final physical X–Y deviation of the workpiece from the marked reference boundary directly represents the planar positioning deviation of the task-space placement point after visual alignment, chassis adjustment, and mechanical execution.

To clarify the optical configuration and control principle used during the manipulation phase, the visual system employs a GC4653 image sensor with a physical pixel size of 2.0 μm. During precision closed-loop alignment, the camera is rigidly mounted approximately perpendicular to the target planar surface, and the mechanical structure constrains the working distance between the camera lens and the target plane to 200 mm. The camera module is equipped with a 3.2 mm F/2.0 lens; according to the manufacturer-specified module parameters, the lens has a field of view of 146° and a distortion below 2%. Although the sensor supports a native resolution of 2560×1440 pixels, the real-time control process operates on 320×240 pixel images. A fixed software lens-correction operation with parameter 1.8 is applied before HSV segmentation and circle fitting. Rather than converting image coordinates into absolute metric coordinates, the system employs an image-based planar closed-loop strategy: the detected center coordinate of the concentric ground marker is continuously transmitted to the low-level controller, where it is compared with a predefined image-plane reference coordinate, and the four Mecanum wheels iteratively adjust the chassis position according to the resulting image-plane offset.

The local alignment follows a two-stage protocol. First, the IMU-based yaw pre-alignment described above adjusts the chassis orientation to the task-specific target angle. Second, after this orientation is reached, the visual controller performs only X–Y translational micro-adjustment. The typical initial planar offset between the detected marker center and the reference coordinate is approximately 5 cm at the physical working plane. For each valid visual-coordinate update, independent X- and Y-axis PID controllers generate translational velocity commands from the corresponding image-plane errors. Both controllers use $K_{p}=0.4$, $K_{i}=0.001$, and $K_{d}=0.05$. The commanded wheel velocities are therefore error-driven and vary throughout the alignment process; the local alignment does not follow a pre-defined constant-velocity or constant-acceleration trajectory.

A 0.8-pixel deadband is applied during fine adjustment. The alignment trial terminates when both X- and Y-coordinate errors remain below 1 pixel for three consecutive valid visual frames, after which the motors are stopped. The manipulator remains locked during this chassis-localization process.

To verify the performance of the visual region localization module, two sets of localization accuracy tests were designed: ROS system stand-alone positioning and vision-aided hybrid positioning. To quantify the localization performance, an end-to-end task-space measurement paradigm was established to evaluate the final 2D planar placement deviation in the X–Y plane. The complete local visual-alignment and physical-measurement protocol is summarized in Table 6.

After closed-loop chassis alignment, the right-side synchronous belt lifting device executes the placement action along the Z-axis to a fixed physical limit, while the mechanical structure constrains the spatial orientation. The manipulator remains locked during the chassis-localization process. Consequently, the quantitative evaluation focuses on the final X–Y planar deviation. The marked physical reference boundary specifies the desired physical placement position and serves as the ground-truth reference for the task-space measurement; the image-plane reference coordinate is a calibrated controller setpoint corresponding to this physical target condition. After each autonomous placement, the absolute planar displacement between the workpiece and the marked physical reference boundary is measured directly using a digital Vernier caliper with a resolution of 0.01 mm. This physical measurement provides the final placement deviation after visual sensing, closed-loop chassis adjustment, and mechanical execution.

The ROS system positioning test (Table 7) utilized LiDAR as the primary sensor and characterized the mobile-base navigation localization baseline. The average translational RMSE and maximum translational deviation were 2.20 cm and 4.60 cm, respectively, while the corresponding rotational RMSE and maximum rotational deviation were 0.86° and 1.83°, respectively. In the worst-case trial, these values increased to 2.80 cm, 5.50 cm, 1.09°, and 2.29°, respectively. These centimeter-level deviations establish the localization baseline before local visual alignment and explain why ROS navigation alone is insufficient for the final precision-handling stage.

To enhance localization precision, vision and gyroscope data are introduced for supplementary calibration. The IMR first adjusts its attitude based on the angular deviation provided by the gyroscope. Subsequently, the vision system identifies the positioning markers in the placement area. After processing via HSV color space conversion, morphological optimization, and Kalman filtering, the data is input into the inverse kinematics equations of the Mecanum wheels to finalize the position and attitude adjustment.

Table 8 presents the end-to-end task-space planar positioning results obtained from 100 independent placement trials for each target color using the proposed visual-servoing method. The statistical results indicate that the Mean Absolute Error (MAE) is consistently maintained around 1.2–1.3 mm. Crucially, the error distribution demonstrates that 95% of the physical X–Y positioning deviations are strictly bounded within the ±2 mm tolerance zone. Even for the extreme outliers, the maximum deviation is constrained to 3.00 mm, reducing the severe long-tail errors present in the baseline method. Furthermore, the low standard deviation (σ<0.65 mm) verifies the trajectory smoothing capability of the Kalman filter. With 95% of tested trials falling within the ±2 mm tolerance, the proposed coarse-to-fine strategy meets the target precision requirement for the evaluated smart-manufacturing scenario.

The results in Table 8 are obtained from end-to-end physical X–Y placement measurements. After each autonomous placement, the final X–Y planar deviation between the workpiece and the marked physical reference boundary is measured using a digital Vernier caliper with a resolution of 0.01 mm. Because the manipulator–base geometry, end-effector orientation, and Z-axis placement limit are constrained during this operation, the measured deviation directly represents the X–Y positioning error of the task-space placement point. Therefore, the reported positioning performance is based on physical task-space deviations rather than on image heatmaps, annotation distributions, or workpiece-recognition metrics. The measured MAE values of 1.21–1.37 mm and standard deviations below 0.65 mm quantify the final task-space planar positioning accuracy of the complete visual–kinematic alignment chain under the tested conditions.

#### 4.6.1. Comparative Analysis: Proposed Algorithm vs. Hough Circle Transform

To verify the robustness of the adaptive visual alignment algorithm proposed in this study under complex industrial environments, comparative experiments were conducted against the classic standard Hough circle transform (SHCT) algorithm.

**Figure 23:**
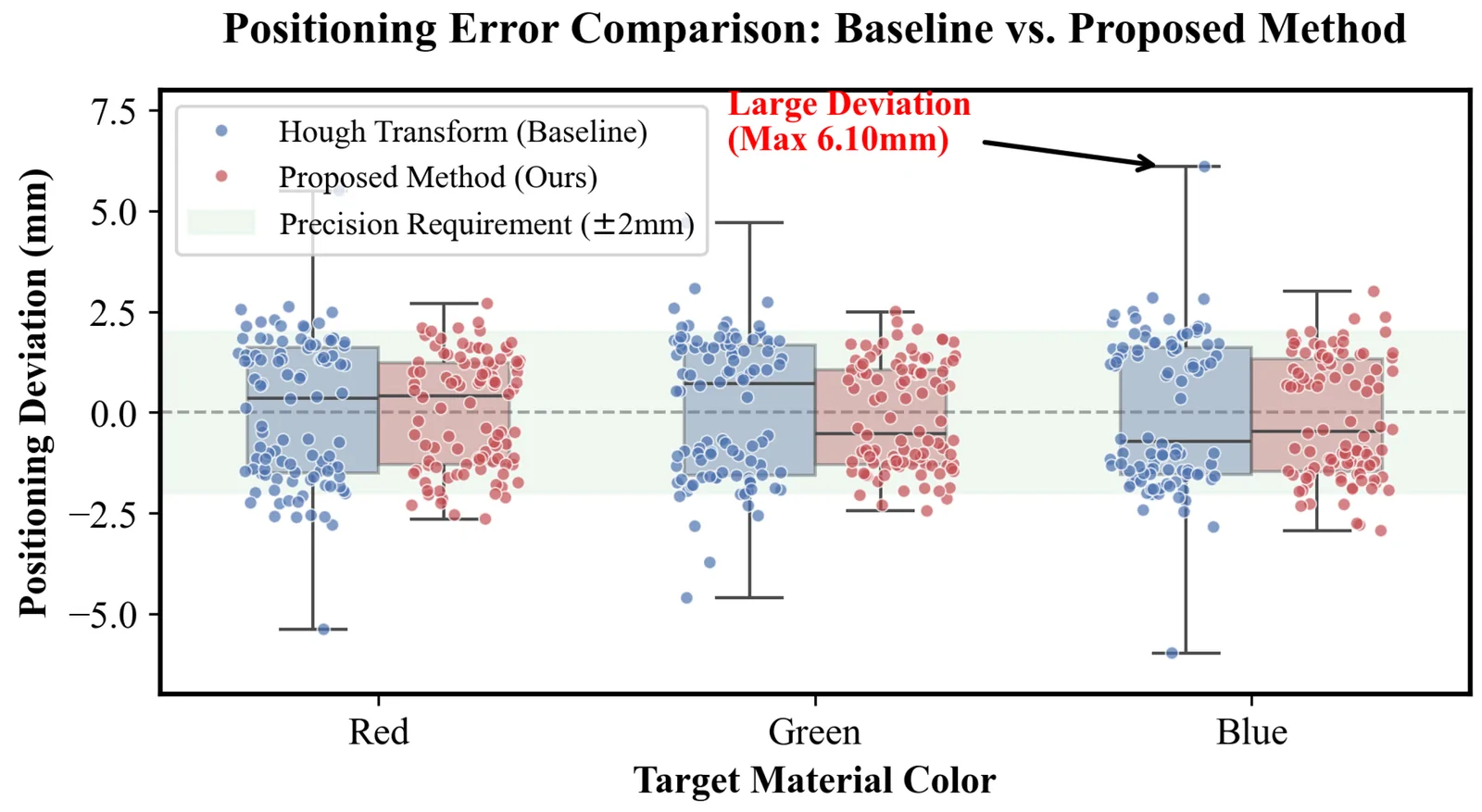
Comparison of position errors showing reduced error spread of the proposed method against the baseline.

As presented in Table 9 and Figure 23, the SHCT algorithm was evaluated over 100 repeated trials for each color condition. The quantitative results show greater error dispersion for the SHCT baseline under the tested environmental conditions:

- **Larger Maximum Deviations:** The maximum absolute localization errors for red, green, and blue targets reached 5.50 mm, 4.70 mm, and 6.10 mm, respectively. In high-precision manufacturing, these larger deviations exceed the ±2 mm tolerance threshold and indicate an increased risk of placement or grasping failure under the tested conditions.
- **Poor Statistical Consistency:** The large standard deviations (σ>0.76 mm across all colors) indicate drastic coordinate jitter. The standard SHCT algorithm lacks robustness against irregular ring edges caused by illumination fluctuations.
- **Conclusion:** The experimental data indicate that the standard SHCT algorithm was less stable in the tested dynamic scenarios, supporting the use of the proposed “coarse-to-fine” adaptive alignment strategy to reduce these errors.

#### 4.6.2. Result Analysis

The experimental data are presented in Table 10. The quantitative relationship between the proposed local visual-processing components and the final positioning performance is established by the physical placement measurements in Table 8 and the module-ablation results in Table 10. Removing morphological processing increases the MAE from 1.21–1.37 mm for the full method to 3.85–4.32 mm and increases the maximum error to 5.92–6.54 mm. This result shows that morphological restoration is required to obtain a reliable geometric center from the discontinuous marker boundary. Removing Kalman filtering leaves the MAE close to the full-method level but increases the standard deviation to 1.38–1.45 mm and the maximum error to 4.55–5.10 mm. This result indicates that the Kalman filter primarily suppresses vibration-induced temporal coordinate fluctuations. The full method combines both functions and produces the physical placement results summarized in Table 8. In addition, the SHCT comparison in Table 9 provides a conventional baseline for the proposed local visual-alignment method.

From the quantitative data in Table 10, the following observations can be made:

1. **Significant Decline in Accuracy:** After removing morphological operations, the average positioning error (MAE) for the three colors increased from approximately 1.2 mm to over 4.0 mm. This is attributed to the physical gaps present in the color rings in the raw images (as shown in Figure 23). Without dilation and closing operations, the binarized image contains disconnected arc segments rather than a closed ring, causing the center position fitted by the smallest enclosing circle algorithm (implemented via cv2.minEnclosingCircle) to deviate from the true geometric center. Conversely, the Ablated (w/o Kalman) group maintains a stable MAE (1.25–1.31 mm), indicating that the filter primarily targets dynamic fluctuations rather than shifting the static geometric baseline.
2. **Deterioration of Trajectory Stability:** The standard deviation of the error in both ablation groups increases significantly compared to the full proposed system. For the Ablated (w/o Morph) configuration, the standard deviation is approximately three times higher (e.g., the green group increased from 0.64 mm to 2.13 mm) due to systemic input noise from contour jumps. Similarly, for the Ablated (w/o Kalman) group, the standard deviation spikes to 1.38–1.45 mm. This demonstrates that in the absence of temporal smoothing, high-frequency mechanical disturbances from the chassis movement and the synchronous belt lifting device directly propagate into the visual feedback, inducing distinct coordinate jitter.
3. **Risk of Extreme Operational Errors:** The maximum absolute localization errors in both ablated configurations easily breach the strict precision threshold required for reliable automation. The Ablated (w/o Morph) group exhibits a maximum error of 6.54 mm due to incomplete features, while the Ablated (w/o Kalman) group records a maximum error of 5.10 mm due to unmitigated physical vibrations. Both scenarios exceeded the allowable tolerance of ±2 mm for industrial precision grasping, indicating that relying solely on binarization or open-loop filtering was insufficient to suppress the worst-case long-tail errors under the tested conditions.

**Figure 24:**
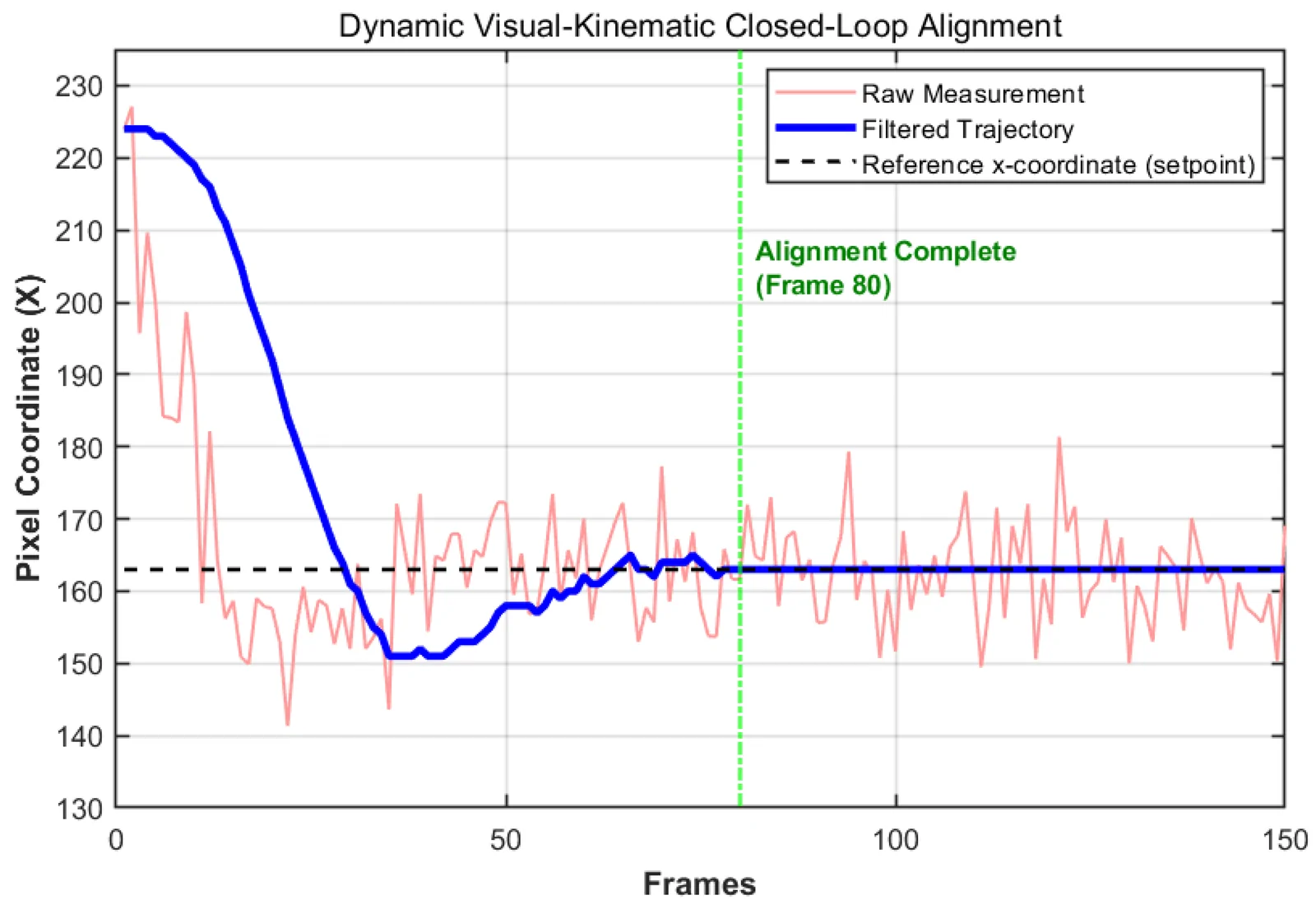
Kalman-filtered visual-kinematic trajectory showing suppressed high-frequency positioning jitter.

To explicitly quantify the dynamic noise-suppression efficacy of the proposed six-dimensional Constant Acceleration (CA) Kalman filter, Figure 24 presents a representative full-cycle visual–kinematic closed-loop alignment trajectory over 150 frames. The red trace denotes the raw image-plane X-coordinate measurements received from the visual module, whereas the blue trace denotes the corresponding Kalman-filtered trajectory. The horizontal dashed line denotes the predefined image-plane reference X-coordinate used as the controller setpoint, rather than an externally measured ground-truth trajectory. The X-coordinate trace is shown as a representative example; the Y-coordinate is processed using the same CA Kalman-filter structure, PID control parameters, and continuous three-frame convergence rule.

During the dynamic approach phase, the raw visual measurements exhibit severe high-frequency coordinate jitter induced by mechanical vibration of the synchronous belt lifting device. Such persistent oscillation can prevent steady-state convergence within the predefined 10 s (150-frame) operational timeout. If the system relied solely on the raw measurements, the unmitigated noise could cause unstable control commands, random trigger failures, or premature timeouts, leading to substantial placement deviations; this effect is consistent with the 4.80 mm maximum error observed for the unfiltered condition in Table 10.

In contrast, the CA Kalman filter suppresses discrete outliers and tracks the underlying physical motion, thereby providing a stable coordinate input to the X–Y PID controller. After the independent IMU-based yaw pre-alignment stage, the visual controller performs only X–Y translational micro-adjustment. Alignment is declared only when both X- and Y-coordinate errors remain below 1 pixel for three consecutive valid visual frames. In the representative trial, the alignment-complete event is marked at Frame 80; the motor commands are then stopped and the alignment routine exits. Figure 24 provides a mechanism-level illustration of coordinate smoothing and closed-loop convergence. The final physical X–Y placement accuracy is evaluated independently by the end-to-end measurements in Table 8 and the module-ablation results in Table 10.

#### 4.6.3. Conclusions

The results of the ablation study indicate that the morphological processing module contributed substantially to the observed improvement in final task-space X–Y positioning accuracy under the tested conditions. It repairs the non-continuous features of industrial markers, providing high-quality geometric input for subsequent contour extraction and filtering algorithms. Kalman filtering and binarization alone do not restore the missing contour features caused by fragmented marker boundaries. Under the tested working conditions, the complete “Vision-Actuation Fusion + Morphological Enhancement” strategy was necessary to achieve the observed precision improvement.

### 4.7. End-to-End Task Workflow and System Robustness

**Figure 25:**
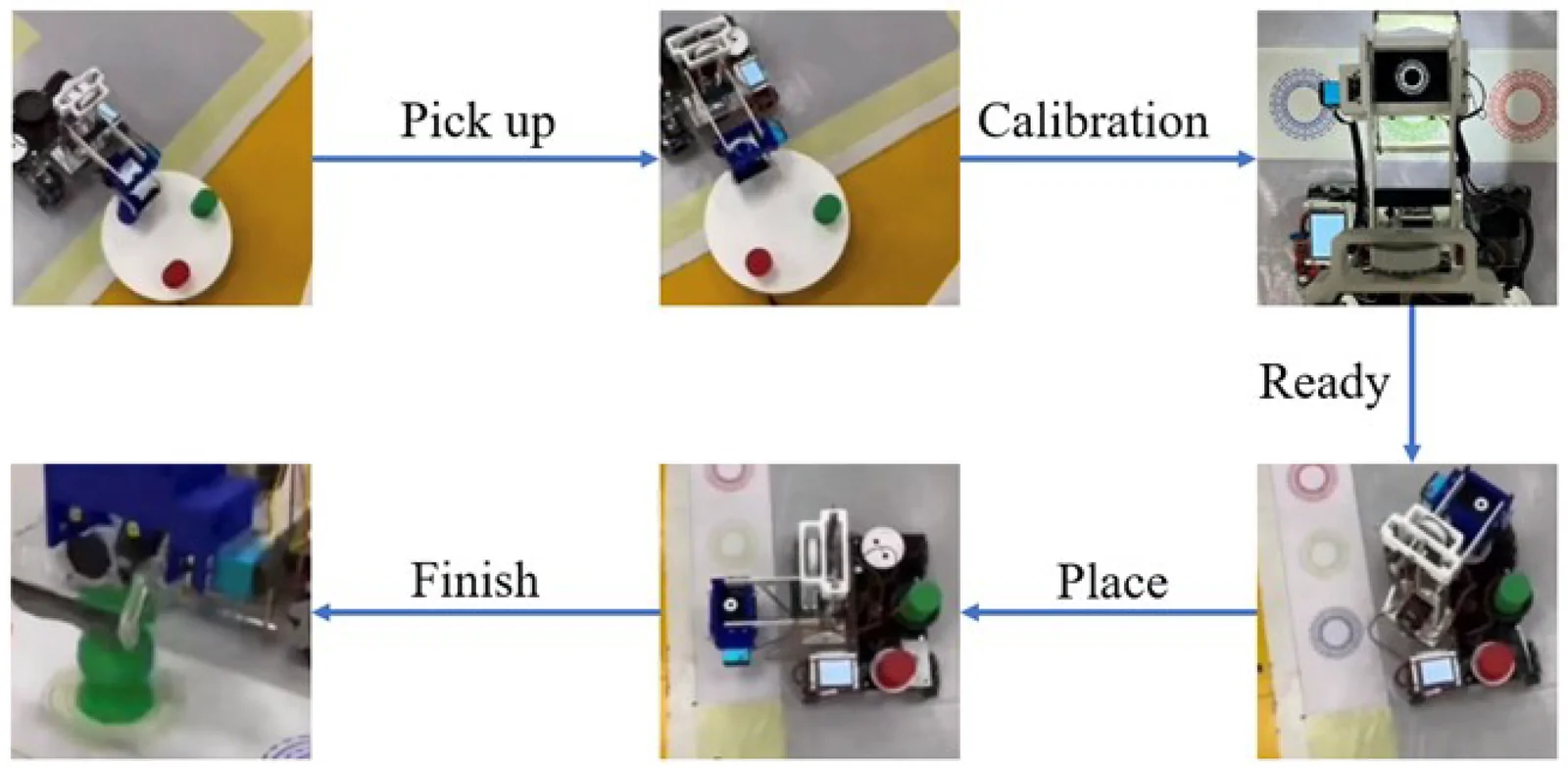
Flowchart of the localization and operation process for closed-loop recognition, grasping, and placement.

The end-to-end task workflow provides an operational scenario for evaluating the proposed coordination mechanism and comprises four key stages: workpiece recognition, precise localization, stable grasping, and designated placement (Figure 25). After the vision system completes the precise localization of the target workpiece, data is transmitted in real-time to the control system to guide the IMR and the robotic arm to adjust their attitude and position. During the grasping process, the vision system continuously tracks the material position, dynamically correcting the motion parameters of the robotic arm and IMR via PID algorithms to ensure grasping stability. Once the material is grasped, the IMR moves to the preset placement area via the ROS navigation stack. The vision system then reuses recognition scheme to achieve precise localization of the placement area, guiding the robotic arm to place the material smoothly.

**Figure 26:**
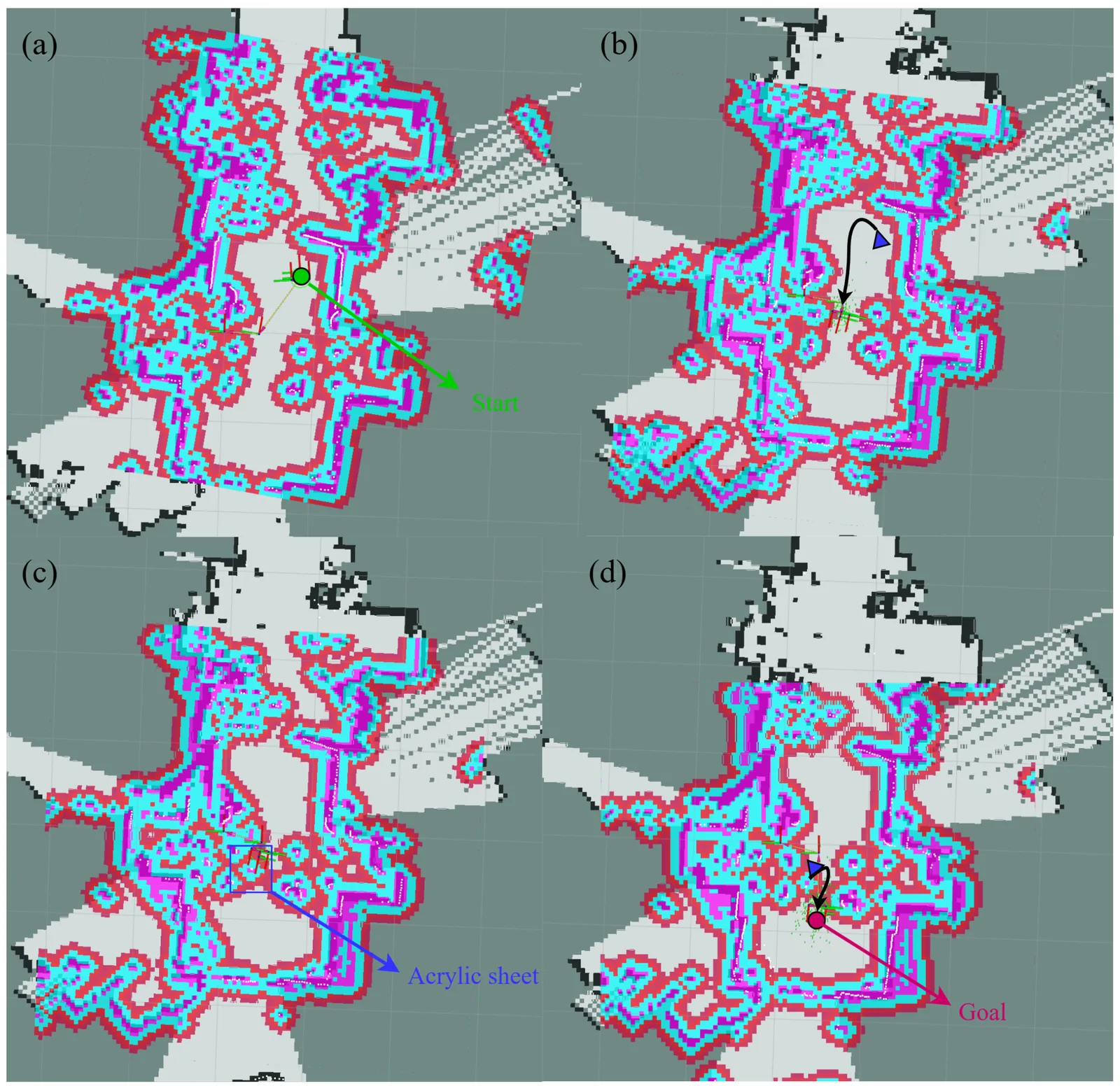
State sequence of transparent-obstacle avoidance in a narrow, obstacle-dense workshop-like layout. (**a**) Initial robot pose and goal command; (**b**) visual preemptive deceleration during the approach; (**c**) ultrasonic obstacle-point observation added to the local costmap near the acrylic sheet; (**d**) local-costmap update, local replanning, and goal-directed motion. Blue triangles denote the robot poses and headings, and black curves denote the representative travelled path.

To make the navigation-safety coordination process explicit within a concrete material-transfer task, Figure 26 presents a representative transparent-obstacle-avoidance run in a narrow, obstacle-dense workshop-like layout. After the host computer issued a navigation goal, the IMR initially faced away from the target and executed a turning maneuver. During the approach, the visual module provided an image-plane early-warning cue and triggered preemptive deceleration before the IMR entered the close-range safety zone. This speed reduction limited the forward distance travelled while the ultrasonic safety response and local-costmap update were being completed. The physical acrylic sheet along the planned route was insufficiently represented in the 2D LiDAR map. At close range, the ultrasonic array detected the obstacle and activated the hardware safety response; the corresponding ultrasonic measurement was subsequently uploaded as an additional point observation in the ROS local costmap. The costmap inflation layer expanded this point observation into a conservative clearance region. In this representative run, preemptive deceleration kept the IMR outside the resulting inflated region when the local costmap was updated, allowing the ROS local planner to generate a curved detour and resume goal-directed navigation. Without this speed reduction, the robot could enter the newly inflated region before replanning is completed, increasing the risk of a local-costmap blockage and recovery requirement in dense layouts. The blue triangles indicate the robot poses and headings, whereas the black curves show the representative travelled path.

The robustness of the system was evaluated under the tested indoor industrial experimental conditions. The system operated stably within an illumination range of 200–1000 lux and maintained continuous tracking performance when the target material was partially occluded, with an occlusion area of up to 30%. These experiments were conducted in an experimental environment designed to reproduce representative industrial operating conditions. Broader generalization to other industrial visual-inspection scenarios requires additional datasets and evaluation.

## 5. Discussion

The experimental results evaluate the proposed decision-level multi-sensor coordination mechanism under the tested baselines and industrial conditions. The evidence is organized according to the different functional contributions of the system. Table 4 evaluates the navigation-level decision policy by progressively activating LiDAR-based geometric replanning, vision-triggered semantic early warning, and ultrasonic near-field protection under matched scenario conditions. Table 7 establishes the centimeter-level positioning baseline provided by the ROS–LiDAR navigation stack. Table 8, Table 9 and Table 10 and Figure 24 independently evaluate the visual–kinematic alignment chain through end-to-end physical X–Y placement measurements, comparison with the standard Hough circle transform, module ablation, and representative raw-versus-filtered coordinate trajectories. Table 1 and Table 2 characterize the edge-deployment feasibility of the visual-perception subsystem in terms of deployment recognition performance, inference speed, and runtime memory consumption. This organization separates the contribution of the decision policy from the characteristics of the individual detector and hardware components, while linking each principal claim to a corresponding quantitative evaluation.

1. A hierarchical obstacle avoidance strategy combining visual recognition and LiDAR ranging reduces false braking events of traditional single-sensor systems, balancing safety and operational efficiency. Quantitatively, at the macroscopic navigation level, the LiDAR-only geometric planning yielded a 62% success rate with 19 collisions (Table 4). Activating the YOLOv5s semantic output within the proposed decision policy provided an image-plane early-warning signal that initiated preemptive deceleration, reducing mid-range collisions and increasing the success rate to 82%. Furthermore, the ultrasonic hardware-interrupt mechanism provided a near-field safeguard against optical sensing failures. Under the tested conditions, the full system recorded no rigid collisions across the 50 trials and achieved a mean response time of 48.2±2.4 ms in the controlled straight-line intrusion test; the navigation success rate was 96%.
2. The visual–kinematic alignment chain achieves high-precision task-space planar positioning under the tested conditions: 95% of the final physical X–Y positioning deviations remain within ±2 mm, meeting the tolerance requirement for the evaluated industrial material-handling task. The complementary roles of these sub-systems were distinctly validated (Table 10): the HSV morphological processing module proved essential for maintaining geometric feature integrity under complex illumination (without it, the MAE of red targets degraded from 1.27 mm to 4.32 mm). Concurrently, for the red-marker condition in the module-ablation experiment, the Kalman filtering module reduced the standard deviation from 1.42 mm in the unfiltered condition to 0.65 mm in the full method.
3. The synergy of the Mecanum wheel chassis, lifting robotic arm, and global localization enables omnidirectional movement and precise grasping, enhancing the system’s passability in narrow spaces.

Meanwhile, the experiments also revealed existing limitations of the system:

1. Under extreme lighting conditions (e.g., direct strong light or illumination <200 lux), the detection accuracy of the YOLOv5s model decreases slightly, which may be related to the model’s insufficient adaptability to lighting changes.
2. The computational complexity of morphological operations and Kalman filtering increases the hardware load on the MaixCAM camera, resulting in a slight drop in frame rate during continuous operation.
3. Vision-aided hybrid positioning relies on the precise calibration of visual sensors and LiDAR; external environmental interference (such as ground flatness, vibration, etc.) may affect calibration accuracy.

These issues will be addressed in future research through algorithm optimization (such as light-adaptive model training) and hardware upgrades (such as high-performance edge computing modules).

## 6. Conclusions

This study formulates and experimentally evaluates a decision-level multi-sensor coordination strategy to bridge the error gap between centimeter-level mobile-base navigation and millimeter-level final planar positioning in industrial mobile-robot tasks. ROS provides the baseline navigation and path-planning functions, while the proposed strategy assigns complementary roles to visual semantic recognition, LiDAR geometric ranging, ultrasonic near-field protection, and local visual–kinematic alignment. These independent sensing outputs are coordinated through explicit decision rules, spatial trigger conditions, and safety priorities, allowing the complete task to be executed on a resource-constrained edge platform without requiring computationally intensive raw-data fusion.

1. **Navigation safety through complementary sensing.** The navigation layer combines visual semantic early warning, LiDAR-based geometric ranging and local replanning, and ultrasonic near-field protection. Visual perception provides an early cue for preemptive deceleration, LiDAR supports geometric navigation, and ultrasonic sensing provides an additional safety response when optical or geometric sensing is insufficient, including the transparent-obstacle condition illustrated in Figure 26. Under the tested conditions, the complete system achieved a 96% navigation success rate with no rigid collisions in 50 trials, and the controlled intrusion test produced a mean hardware-response time of 48.2±2.4 ms. These results support the effectiveness of assigning complementary sensing roles through decision-level coordination.
2. **Coarse-to-fine planar positioning.** The ROS–LiDAR navigation stack provides the centimeter-level mobile-base positioning baseline, while local visual–kinematic alignment corrects the residual error before final placement. HSV-based morphological restoration improves the recovery of the geometric marker center, and Kalman filtering suppresses vibration-induced temporal jitter during visual servoing. In the end-to-end placement trials, 95% of the final physical X–Y positioning deviations remained within ±2 mm under the tested conditions. The ablation results further showed that removing morphological restoration increased the red-target MAE from 1.27 mm to 4.32 mm, whereas removing Kalman filtering increased the corresponding standard deviation from 0.65 mm to 1.42 mm. These results indicate that the local visual–kinematic stage complements ROS–LiDAR navigation by providing the final planar positioning accuracy required by the evaluated material-handling task.
3. **System-level implementation on a resource-constrained platform.** The MaixCAM–STM32 implementation connects navigation, local alignment, grasping, and placement within one executable workflow. The selected YOLOv5s model serves as an auxiliary semantic-perception module and achieved an average deployed inference speed of 24.5 FPS, providing the accuracy–speed–memory trade-off required by the implemented hardware architecture. This result demonstrates the practical feasibility of the complete coordination strategy on the evaluated platform; it is not intended as a universal ranking of all object-detection architectures.

The findings are limited by the evaluated hardware, datasets, and indoor industrial experimental conditions. Detection confidence may decrease under extreme illumination below 200 lux, and the computational load of the cascaded visual-processing modules may cause minor frame-rate fluctuations. Final positioning also remains sensitive to calibration accuracy, ground unevenness, and strong mechanical disturbances. Future work will investigate illumination adaptation and online recalibration and will evaluate newer detector architectures after an upgrade of the edge-computing hardware to provide sufficient memory capacity and NPU throughput.

## Figures and Tables

**Table 1:** Edge inference performance comparison of YOLOv5s, YOLOv8s, and YOLOv11s on MaixCAM.

Metric	YOLOv5s (INT8)	YOLOv8s (INT8)	YOLOv11s ^*b*^
Deployment mAP@0.5 (INT8)	62.5%	63.8%	N/A
Inference Speed (FPS)	24.5 FPS	13.2 FPS	N/A
Total Pipeline Latency	40.8 ms	75.7 ms	N/A
– Image Capture	3.0 ms	3.0 ms	N/A
– Pre-processing (Resize)	4.2 ms	4.2 ms	N/A
– Inference (Deployment)	21.5 ms	44.8 ms	N/A
– Post-processing (NMS)	12.1 ms	23.7 ms	N/A
Runtime Memory Consumption	116 MB	168 MB	>256 MB
Real-time Status ^*a*^	YES (>20 FPS)	NO (<20 FPS)	Not deployable
Real-time criterion (>20 FPS)	Met	Not met	Not applicable

^*a*^ Real-time performance on the MaixCAM is defined as an inference speed exceeding 20 FPS. ^*b*^ YOLOv11s could not complete deployment because its runtime memory requirement exceeded the available 256 MB memory of the MaixCAM platform.

**Table 2:** Quantitative comparison between the baseline and edge-optimized YOLOv5s deployment models.

Metric	Baseline YOLOv5s (Standard INT8)	Edge-Optimized Deployment Model
Deployment mAP@0.5 (INT8)	64.1%	62.5%
Parameters	7.02 M	4.49 M
Model Size	14.1 MB	4.8 MB
Runtime Memory Consumption	182 MB	116 MB
Inference Speed	11.2 FPS	24.5 FPS

**Table 3:** Quantitative emergency-braking characteristics in the controlled acrylic-panel intrusion test (N=20, $v_{0}=0.50$ m/s).

Metric Category	Evaluation Metric	Mean ± SD	Worst Case	Measurement Basis
Timing	Trigger-to-command delay ($t_{\mathrm{cmd}}-t_{0}$)	8.5±0.8 ms	9.8 ms	STM32 1 kHz hardware timer
	Command-to-zero-velocity braking duration ($t_{1}-t_{\mathrm{cmd}}$)	39.7±2.1 ms	43.2 ms	Timer input capture of the final encoder edge and no-pulse confirmation
	Total emergency-stop delay ($\Delta t=t_{1}-t_{0}$)	48.2±2.4 ms	52.1 ms	Direct calculation for each trial
Dynamics	Physical braking distance	1.42±0.08 cm	1.58 cm	Time integration of encoder-derived velocity trajectory
	Residual velocity at the declared stopping endpoint	0.00 m/s in all trials	0.00 m/s	Hall-encoder feedback and zero-velocity termination criterion
Clearance	Final physical clearance after stopping	28.6±0.1 cm	28.4 cm	Steel-ruler measurement from the front rigid reference point to the acrylic panel

**Table 4:** System-level navigation performance under varying layouts, densities, illuminations, and velocities (N=50).

Configuration	Success Rate (%)	Collisions	Min Safety Distance (m)	Completion Time (s)	Recovery Time (s)
LiDAR-only	62	19	0.00	48.5	9.2
Vision + LiDAR	82	9	0.00	39.8	6.8
Full System (Proposed)	96	0	0.18	34.2	1.8

**Table 5:** One-factor sensitivity evaluation of the two action-priority thresholds under the full-system configuration (N=50 per setting).

(a) Ultrasonic emergency threshold sensitivity ($D_{\mathrm{vis}}$ = 1.5 m)					
Threshold (m)	Autonomous success (%)	Rigid collision (%)	Deadlock/manual recovery (%)	Minimum noncontact clearance (m)	Completion time (s)
0.20	84	16	0	0.08	33.8
0.30 (baseline)	96	0	4	0.18	34.2
0.40	96	0	4	0.26	41.5
(b) Vision-related upper decision-boundary sensitivity ($D_{\mathrm{ultra}}$ = 0.3 m)					
Threshold (m)	Autonomous success (%)	Rigid collision (%)	Deadlock/manual recovery (%)	Minimum noncontact clearance (m)	Completion time (s)
1.0	80	6	14	0.12	31.1
1.5 (baseline)	96	0	4	0.18	34.2
2.0	98	0	2	0.21	43.6

The three outcome categories are mutually exclusive and collectively exhaustive for each 50-trial setting. Completion time was calculated only from autonomous-success trials. Minimum noncontact clearance was calculated only from trials without physical contact.

**Table 6:** Local visual-alignment and physical-measurement protocol.

Item	Specification
Yaw pre-alignment sensor	HWT101 single-axis gyroscope; Z-axis angle measurement with manufacturer-specified accuracy of 0.1°.
Yaw correction and criterion	Fixed zero-drift correction AdjYaw; IMU-PID yaw alignment to a task-specific target angle; final absolute yaw error ≤0.2°.
Visual sensor and geometry	GC4653 sensor with 2.0 μm pixels; 3.2 mm F/2.0 lens; 146° field of view; distortion below 2%; camera mounted approximately perpendicular to the target plane at a working distance of 200 mm.
Visual input and preprocessing	320 × 240 image input; fixed lens-correction parameter of 1.8; HSV segmentation, morphological restoration, and six-dimensional CA Kalman filtering.
Initial translational condition	Typical initial X–Y offset of approximately 5 cm between the marker center and the image-plane reference coordinate.
X–Y control	Independent image-error-driven PID controllers: $K_{p}=0.4$, $K_{i}=0.001$, and $K_{d}=0.05$ for both axes; Mecanum-wheel commands are updated according to the current visual error.
Motion profile	Low-speed adaptive translational micro-adjustment; no pre-defined constant-velocity or constant-acceleration trajectory.
Termination criterion	0.8-pixel fine-adjustment deadband; both X- and Y-coordinate errors remain below 1 pixel for three consecutive valid visual frames.
Test and measurement	Red, green, and blue marker conditions; 100 independent placement trials per color; illumination range of 200–1000 lux; final X–Y placement deviation measured using a digital Vernier caliper with 0.01 mm resolution.
Representative trajectory	A representative 150-frame raw and filtered image-plane X-coordinate trace is used; the Y-coordinate follows the same filtering and control rules.

**Table 7:** Statistical deviation of ROS system positioning accuracy (N=50).

Metric	Trans. RMSE (cm)	Trans. Max (cm)	Rot. RMSE (°)	Rot. Max (°)
Average (N=50)	2.20	4.60	0.86	1.83
Worst-case	2.80	5.50	1.09	2.29

**Table 8:** Statistical deviation of HSV visual positioning accuracy (N=100 per color).

Color Condition	MAE (mm)	Std. Dev.	Max Error (mm)
Red	1.27	0.65	2.70
Green	1.21	0.64	2.50
Blue	1.37	0.63	3.00

**Table 9:** Statistical deviation of SHCT positioning accuracy (N=100 per color).

Color Condition	MAE (mm)	Std. Dev.	Max Error (mm)
Red	1.58	0.87	5.50
Green	1.57	0.77	4.70
Blue	1.64	1.04	6.10

**Table 10:** Comparison of positioning accuracy in module ablation study (N=100).

Target Color	Method	MAE (mm)	Std Dev (mm)	Max Error (mm)	Observation
Red	Ablated (w/o Morph)	4.32	1.87	6.54	Drastic jitter of center
	Ablated (w/o Kalman)	1.25	1.42	4.80	High-frequency coordinate jitter
	Proposed (Full)	1.27	0.65	2.70	Full contour, stable center
Green	Ablated (w/o Morph)	3.85	2.13	5.92	Jumping fitting radius
	Ablated (w/o Kalman)	1.28	1.38	4.55	Severe dynamic oscillation
	Proposed (Full)	1.21	0.64	2.50	Precise fitting, no obvious noise
Blue	Ablated (w/o Morph)	4.15	1.95	6.10	Jumping fitting radius
	Ablated (w/o Kalman)	1.31	1.45	5.10	Continuous trajectory noise
	Proposed (Full)	1.37	0.63	3.00	Smooth alignment

## Data Availability

The original datasets are not publicly available and cannot be shared upon request because they contain identifiable backgrounds of operational industrial facilities, equipment layouts, and task configurations subject to confidentiality and data-security restrictions. To support methodological reproducibility, the manuscript provides the dataset collection and annotation procedures, data splits, camera configuration, experimental conditions, and evaluation protocols. A reference implementation of the local visual–kinematic alignment module described in Section 3.3 and Section 4.6, including HSV-based marker extraction, morphological restoration, six-state Kalman filtering, X–Y PID alignment, and yaw pre-alignment, is publicly available at https://github.com/Acknowing/IMR-local-visual-kinematic-alignment/tree/main/IMR-local-visual-kinematic-alignment (accessed on 2 September 2026).
